# Pan-cell death-related signature reveals tumor immune microenvironment and optimizes personalized therapy alternations in lung adenocarcinoma

**DOI:** 10.1038/s41598-024-66662-1

**Published:** 2024-07-08

**Authors:** Linzhi Han, Jingyi He, Hongxin Xie, Yan Gong, Conghua Xie

**Affiliations:** 1 Department of Radiation and Medical Oncology, Zhongnan Hospital of Wuhan University, 169 Donghu Road, Wuhan, 430071 Hubei China; 2 Tumor Precision Diagnosis and Treatment Technology and Translational Medicine, Hubei Engineering Research Center, Zhongnan Hospital of Wuhan University, 169 Donghu Road, Wuhan, 430071 Hubei China; 3 Hubei Key Laboratory of Tumor Biological Behaviors, Zhongnan Hospital of Wuhan University, Wuhan, China

**Keywords:** Cell death, Lung adenocarcinoma, Immune microenvironment, Immunotherapy response, Radiotherapy response, Cancer, Computational biology and bioinformatics

## Abstract

This study constructed a comprehensive analysis of cell death modules in eliminating aberrant cells and remodeling tumor microenvironment (TME). Consensus analysis was performed in 490 lung adenocarcinoma (LUAD) patients based on 4 types of cell death prognostic genes. Intersection method divided these LUAD samples into 5 cell death risk (CDR) clusters, and COX regression analysis were used to construct the CDR signature (CDRSig) with risk scores. Significant differences of TME phenotypes, clinical factors, genome variations, radiosensitivity and immunotherapy sensitivity were observed in different CDR clusters. Patients with higher risk scores in the CDRSig tended to be immune-excluded or immune-desert, and those with lower risk scores were more sensitive to radiotherapy and immunotherapy. The results from mouse model showed that intense expression of the high-risk gene PFKP was associated with low CD8+ T cell infiltration upon radiotherapy and anti-PD-L1 treatment. Deficient assays in vitro confirmed that PFKP downregulation enhanced cGAS/STING pathway activation and radiosensitivity in LUAD cells. In conclusion, our studies originally performed a comprehensive cell death analysis, suggesting the importance of CDR patterns in reprogramming TME and providing novel clues for LUAD personalized therapies.

## Introduction

Lung adenocarcinoma (LUAD) is the major pathological phenotype of lung cancer with the highest lethal rate globally. Surgical resection, radiotherapy (RT), chemotherapy, targeted medicines and immunotherapy are the main strategies for LUAD patients. Among them, RT is the indispensable one widely used in routine treatment. Almost 77% LUAD patients were subjected to RT, which induced double DNA strand destruction in tumor cells, followed by immunogenic cell death (ICD)^1^. Immunotherapy, especially immune checkpoint inhibitors (ICIs), obtained outstanding achievements in recent years. ICIs such as PD-1, PD-L1 and CTLA-4 antibodies reversed inactivated cytotoxic T cells and enhanced cancer-eradicating immunity. Due to the abundant T cell infiltration in LUAD microenvironment and 40% LUAD patients with PD-L1 positive expression, LUAD patients are more likely to benefit from ICIs^2^. Recently, emerging evidence illustrated that combination of RT and ICIs revolutionized cancer treatment, improving LUAD patients’ survival compared with individual treatment^3–5^.

Theoretically, RT not only led to ICD in regional lesion, but also aroused systemic immune responses that damaged metastatic and nonirradiated neoplasm (abscopal effect)^4^. Moreover, ICIs identified tumor-associated antigens from RT-elicited ICD and further strengthened immunology. However, the majority of LUAD patients with synergic therapies still failed to show satisfactory clinical outcome^6^. Investigating the underlying molecular mechanisms and identifying patients with potential responses to mono-therapy or combined therapies would improve LUAD clinical outcome.

Evaluation of curative effects for surgical resection, RT, chemotherapy and targeted drugs was based on cancer regression or disappearance. With the emergence of promising clinical outcome from immunotherapy, scientists started to focus on cancer cells undergoing ICD. Cancer-related cell deaths consist of non-immunogenic death and ICD. The former, such as apoptosis, leads to the elimination of cancer cells without eliciting lasting immunity^7^, while the latter was involved in the emission of damage-associated molecular patterns (DAMPs) to recruit antigen presenting cells, provoking tumor-specific adaptive immunity (also known as immune-surveillance) and maintaining long-term anti-tumor immunity^8^. ICD is both a friend and a foe in anti-tumor immunity with both pro- and anti-tumor effects^7,9^. Therefore, patients with tumor regression could transiently benefit from clinical therapies, but a considerable proportion of them suffer from tumor recurrence or dissemination^10–12^. There is a high need to identify cell death molecules for eliminating neoplasm, stimulating systemic immunity, inhibiting tumor growth and prolonging LUAD patient survival.

In this study, we originally used consensus clustering method to construct 5 subtypes by analyzing 4 cell death ways (apoptosis, ferroptosis, pyroptosis and necroptosis). The 5 subtypes were significantly related with RT and immunotherapy sensitivity, synergistic responses, tumor stemness, tumor mutation burden (TMB) and copy number variation (CNV). Furthermore, high risk of cell death subtype led to “immune desert” and “immune exclusion” with the decrease of major histocompatibility complex (MHC) class I. According to the 5 subtypes, we established an 8-gene signature to validate accuracy of subtypes. We confirmed that our cell death risk (CDR) subtypes might serve as a reliable predictive metric for RT or/and immunotherapy sensitivity.

## Results

### Identification of 4 types cell death genes

**Figure 1:**
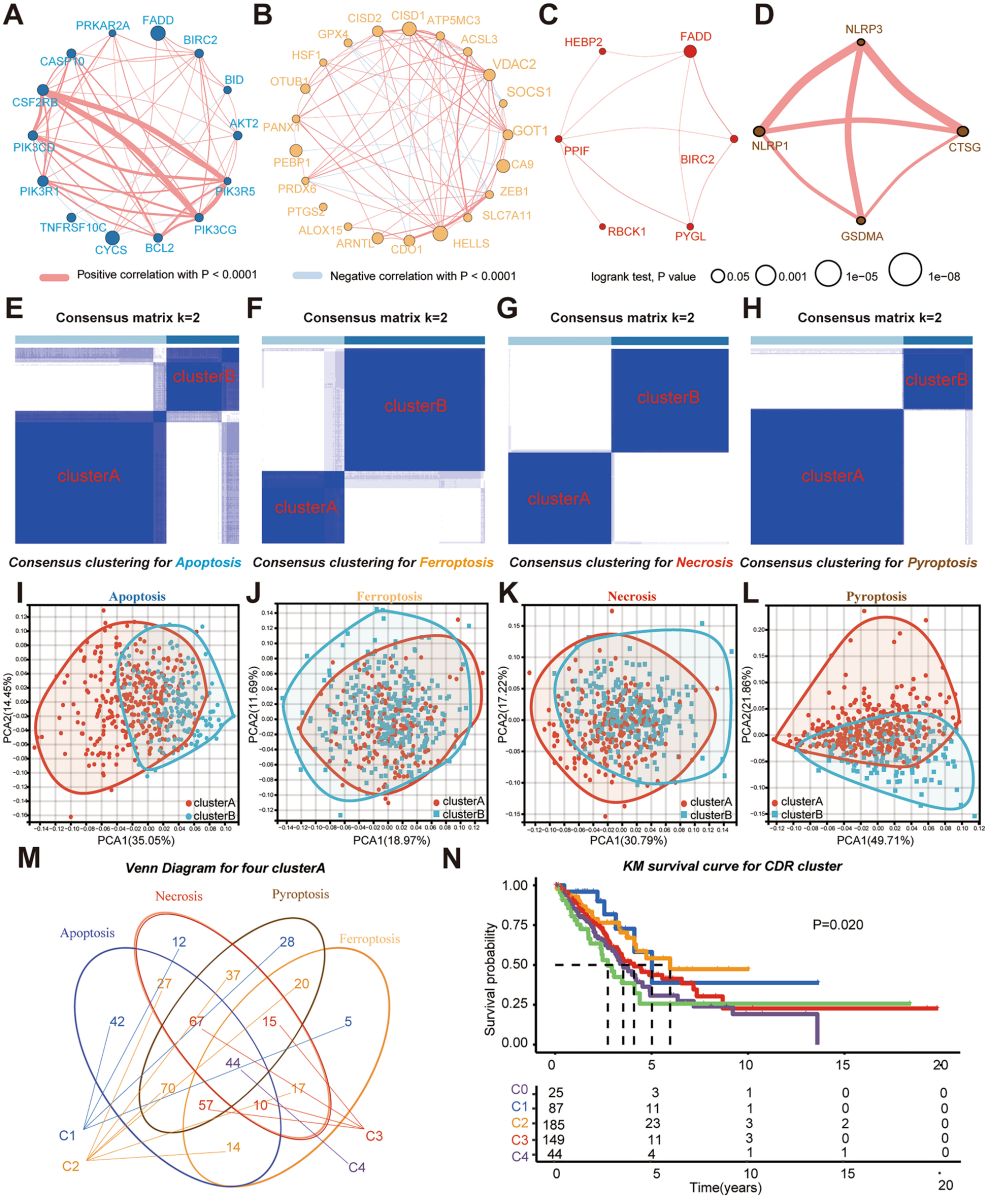
Identification of cell death risk clusters. (**A**–**D**) Prognostic genes from apoptosis (**A**), ferroptosis (**B**), necroptosis (**C**), and pyroptosis (**D**). Thickness of lines represent correlation between genes. (**E**–**H**) Consensus clustering of LUAD patients by apoptosis (**E**), ferroptosis (**F**), necroptosis (**G**), and pyroptosis (**H**). (**I**–**L**) PCA for the 4 cell death patterns. M, identification of CDR via taking intersection with the cell death cluster A in upset plot. N, survival analysis among cluster A stratified by CDR clusters.

We collected CDR genes from published data (Supplementary Table 1), in which genes-related with overall survival were selected through univariate multivariate COX analysis for further analysis (Fig. 1A–D). Patients from the LUAD TCGA database were stratified into cluster A and B using consensus cluster analysis based on CDR genes (Fig. 1E–H). Principal component analysis (PCA) was used to verify the reliability of consensus cluster analysis (Fig. 1I–L). For all the 4 cell death patterns, cluster B had better prognosis (Supplementary Fig. 2A–D) and cluster A with worse prognosis. Based on their cluster A status in the 4 cell death groups, patients were classified into cluster 0 (0 “cluster A” in the 4 cell death groups), cluster 1 (1 “cluster A” in the 4 cell death groups), cluster 2 (2 “cluster A” in the 4 cell death groups), cluster 3 (3 “cluster A” in the 4 cell death groups), cluster 4 (4 “cluster A” in the 4 cell death groups) (Fig. 1M–N). The close correlation between CDR clusters and genes was investigated in composite thermogram (Supplementary Fig. 3A). For clinical characteristics, CDR clusters were notably associated with stage, T stage, N stage (Supplementary Fig. 3B). Similar results were also observed in univariate and multivariate COX analysis, which revealed the tight correlation between CDR clusters and stages (Supplementary Fig. 3C).

### Variation of TME and immunotherapy responses in CDR clusters

**Figure 2:**
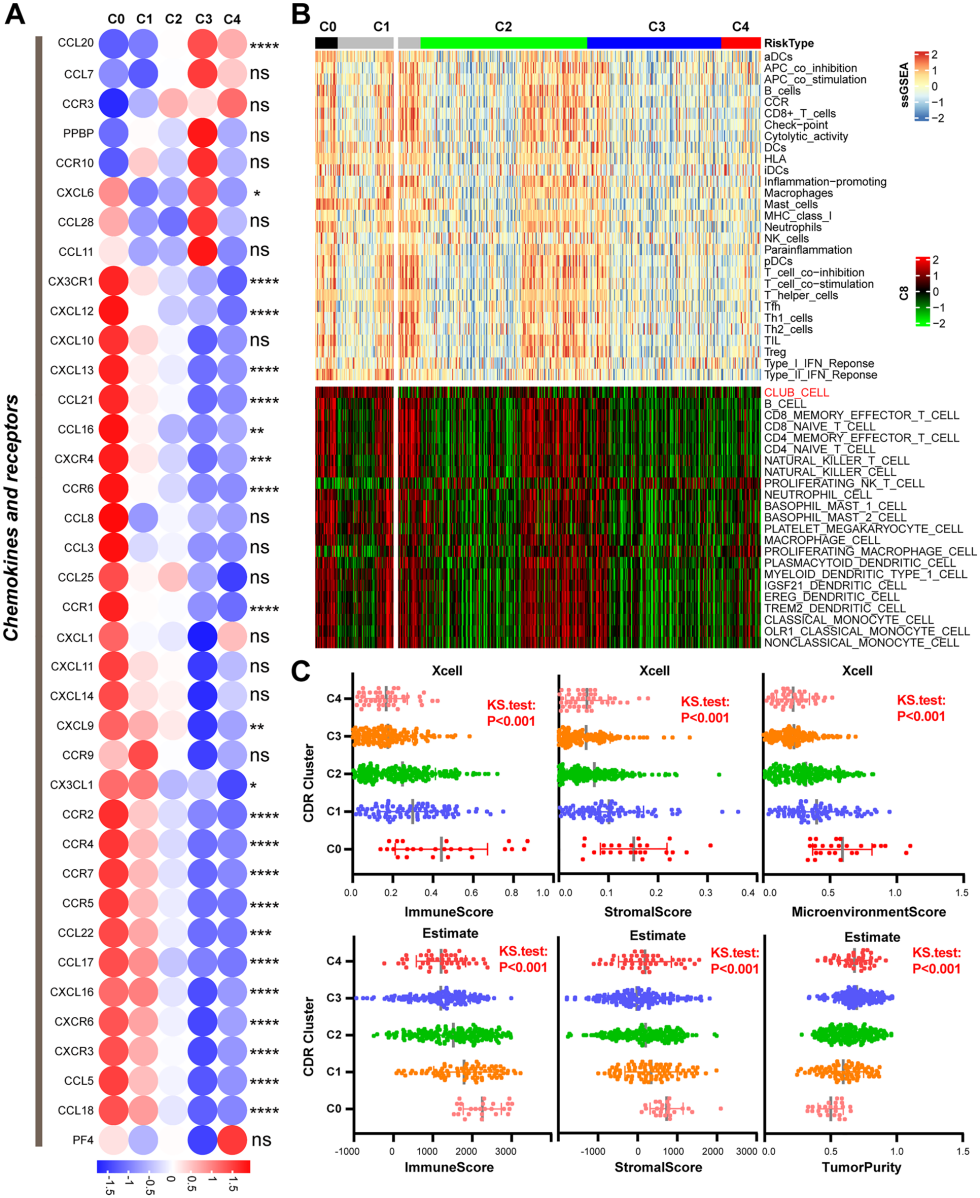
Immune infiltration and immune-associated pathways in CDR clusters. (**A**) Variance of immune-related chemokines/receptors in CDR clusters. (**B**) An overview of immune cells infiltration in CDR clusters. (**C**) TME score among CDR clusters using Xcell, ESTIMATE method. (*, *P* < 0.05; **, *P* < 0.01; ***, *P* < 0.001; ****, *P* < 0.0001; ns, *P* > 0.05).

CD8+ T cells, T helper 1 (Th1) cells, dendritic cells (DCs) and natural killer (NK) cells are the main cells exerting anti-cancer ability in TME. The levels of chemokine receptors and chemokines (CXCR3, CCR6, CXCR4, CCL5, CXCL6 and CXCL12) were higher in the C0 vs. C4 group, which enabled anti-tumor cells migrate to tumor lesions (Fig. 2A, Supplementary Fig. 4)^13^. Patients in the C4 vs. C0 group had less immune cell infiltration and cytokines (Fig. 2B, Supplementary Fig. 4A–C, Supplementary Table 2, 3). Based on cell type signature gene sets (MSigDB Collections, C8), we calculated the abundance of various cell types in LUAD using ssGSEA method. Interestingly, most of cell types had significantly difference in the 5 CDR patterns. Club cells and NK-T cells exhibited obvious decreasing trend in the C0 versus C4 group (Fig. 2B, Supplementary Fig. 4B). Patients in the C0 group had higher immunescore, stromalscore, microenvironmentscore than other ones using Xcell and ESTIMATE method (Fig. 2C). The levels of immune checkpoints, CD8 effectors and angiogenic markers were higher in the C0 group (Supplementary Fig. 4D, Supplementary Table 4). DNA damage repair, DNA replication, nucleotide excision repair, homologous recombination repair and mismatch repair were more active in the C0 vs. C4 group.

**Figure 3:**
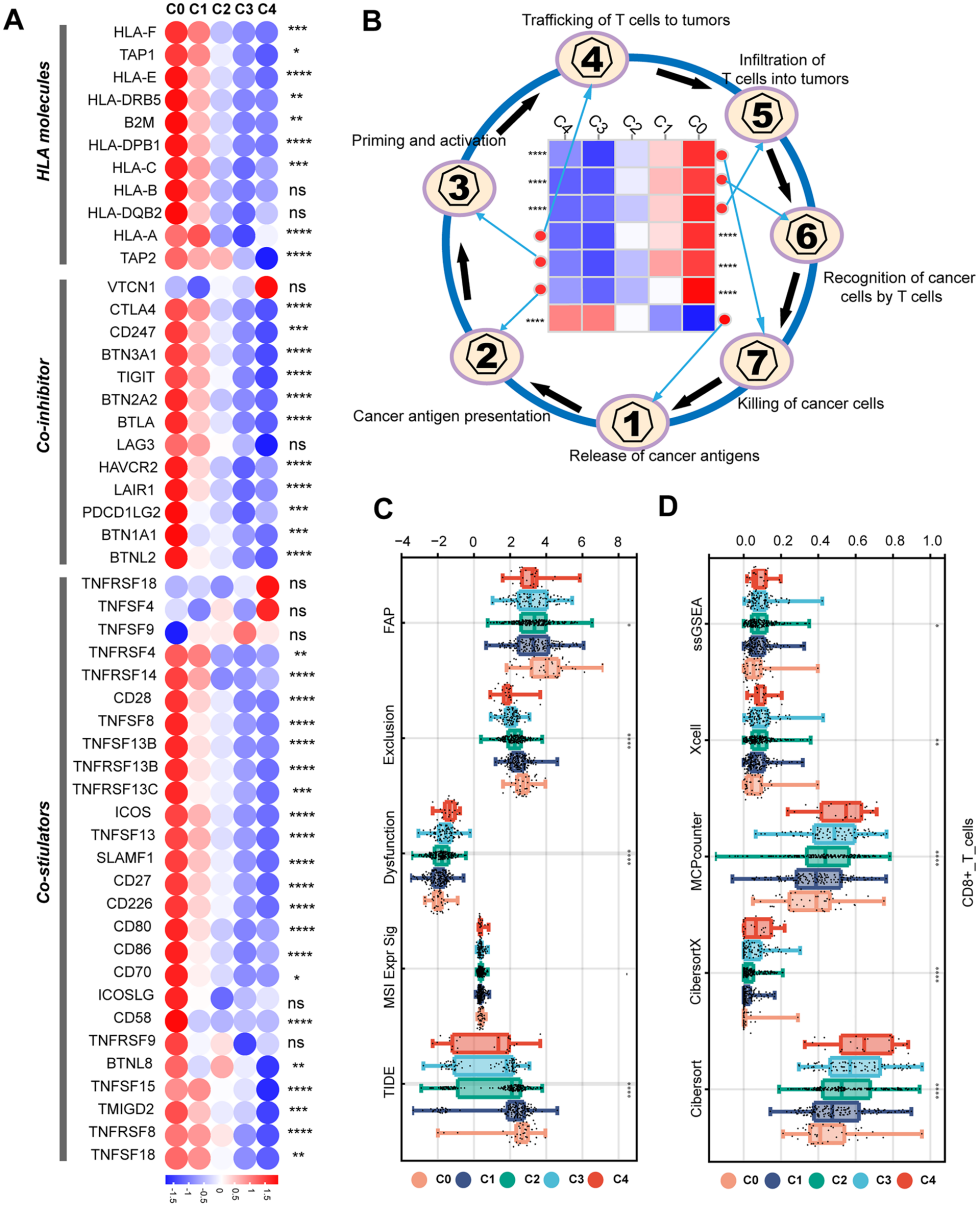
Tumor-immunity cycle and immunotherapy response variation among CDR clusters. (**A**) Expression of leukocyte antigens, immune co-inhibitors and co-stimulators in CDR clusters. (**B**) Cancer-immunity circle difference. (**C**–**D**) Immune scores of CDR clusters using TIDE, MCPCounter, CIBERSORT, CIBERSORTx, ssGSEA. (*, *P* < 0.05; **, *P* < 0.01; ***, *P* < 0.001; ****, *P* < 0.0001; ns, *P* > 0.05).

The expression of human leukocyte antigen (HLA) molecules (MHC family molecules), co-inhibitors (CTLA-4, CD274/PD-L1, TIGIT, HAVCR2), co-stimulators (CD80) are higher in the C0 group than other clusters (Fig. 3A). HAVCR2 was reported to be involved in the process of ICIs re-initiating the tumor antigen-activating effector CD8+ T cells, lacking of which would cause the failed response to ICIs in patients^14^. Therefore, CD8+ T cells with HAVCR2 downregulation in the C1, C2, C3 and C4 might be the potential reason for low immunotherapy response. Moreover, the C0 group had higher cancer antigen presentation, T cells priming and activation, trafficking of T cells to tumors, T cell infiltration in TME, recognition of cancer cells by T cells, cancer cells killing ability in physical eliminating cancer cells process (Fig. 3B and Supplementary Table 5). Furthermore, TIDE analysis suggested that the C0 group had lower TIDE scores than other clusters, implying that these patients would be more likely to benefit from ICIs. In addition, patients in the C4 group intended to have immune cell exclusion and increase of FAP (a signal of thick matrix around tumor and hindering CD8+ T lymphocyte infiltrates) (Fig. 3C)^15,16^. There was a downregulation of CD8+ T cell infiltration from CDR C0 to C4 group (Fig. 3D). These would contribute to the bad prognosis of patients in the C4 group.

### Correlation between CDR patterns and RT sensitivity

According to previous advances^1^, we found radiotherapy sensitive (RS) and prognosis-related genes in LUAD patients (Supplementary Fig. 5A). After consensus clustering analysis, patients were ranked into the radiotherapy cluster 1(RC1) and radiotherapy cluster 2(RC2) groups (Supplementary Fig. 5B), the verification of which was in Supplementary Fig. 5C. Survival curve was used as a tool to identify RS and radiotherapy resistant (RR) groups. The patients in RC2 group had longer survival than those in the RC1 group. As a result, RC2 cluster was named as the RS group and RC1 was reckoned as the RR one (Supplementary Fig. 5D). Moreover, the GSEA revealed that oxidative phosphorylation and citrate cycle pathway were enriched in RR group. Apoptosis, primary immunodeficiency and cell adhesion molecules cams were enriched in RS group (Supplementary Fig. 5E,F, Supplementary Table 6). CDR C0 was more likely to benefit from RT, for the significant increase from RR to RS group. The RS group had lower stage than the RR group (Supplementary Fig. 5G).

### Association of CDR phenotypes with tumor stemness

The difference of tumor stemness was next investigated in the 5 CDR clusters. Our results evidenced that the expression of cancer stem cell surface markers, including EPCAM, CSF3R, CD34, CD96 and ENG, were significantly different (Supplementary Fig. 6A, Supplementary Table 7). We further compared the tumor stemness index such as mRNAsi, mDNAsi, EREG-mDNAsi and DMPsi in the CDR clusters. From the C0 to C4 groups, the tumor stemness index showed a significant upward trend (Supplementary Fig. 6B). Similarly, a higher tumor stemness index was found in the RR group. Moreover, the analysis of the top 10 tumor signals showed that the cell cycle and PI3K signals were significantly activated during the increase of high-risk events of cell death, and the NOTCH, RAS and TGF-beta signals were evidently suppressed (Supplementary Fig. 6C, Supplementary Table 8).

### Differences in genome characteristics of the 5 CDR clusters

**Figure 4:**
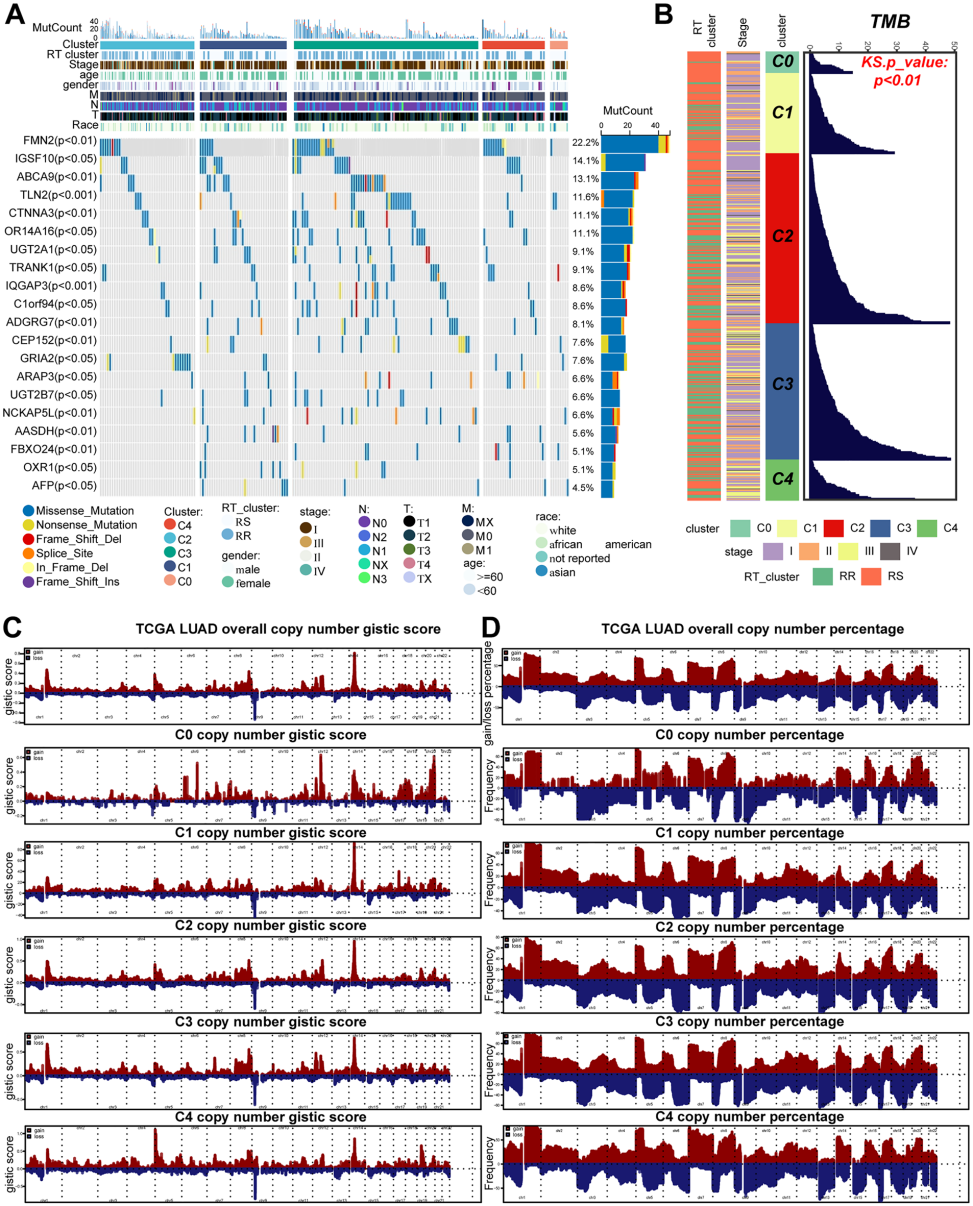
Genome variation map in CDR clusters. (**A**) Heatmap of correlation among clinical factors, CDR clusters and 20 RS mutation genes of LUAD patients. (**B**) Difference of tumor burden, stage and RT cluster in CDR clusters. (**C**–**D**) Frequencies of CNVs and percentage of CDR clusters in TCGA-LUAD cohort patients. Gain (brown) or loss (blue) autosomes of LUAD patients from the TCGA-LUAD cohort.

**Figure 5:**
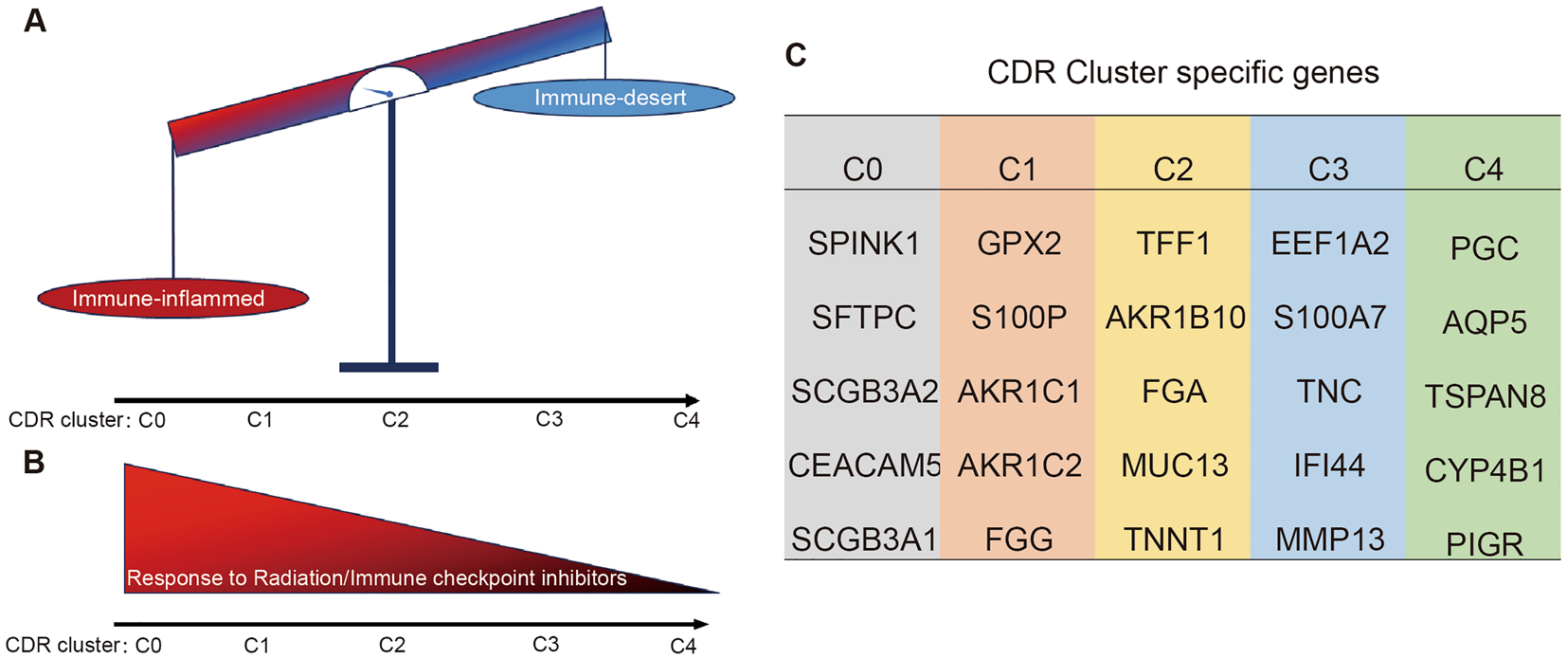
The characteristics of TME features (**A**), treatment response (**B**), and top 5 highly expressed genes (**C**) in each CDR cluster.

The waterfall chart displayed the top 20 genes with the highest mutation frequency in the TCGA-LUAD cohort. The mutation frequency of TLN2, CTNNA3 and IQGAP3 exhibited a significant difference in CDR phenotypes (Fig. 4A). TP53, KRAS, KEAP1, STK11 and EGFR are common driver mutations in LUAD. Among them, EGFR mutations were statistically significant between our five CDR clusters (*p* < 0.01, Supplementary Fig. 6D), suggesting that EGFR mutations might influence cell death. Across the board, TMB presented a remarkable increasing trend from the C0 to C4 groups (Fig. 4B). Consistently, high TMB was more likely to be associated with the RR group. The copy number GISTIC score (Fig. 4C) and the copy number percentage (Fig. 4D) of the gain (dark red) and loss (dark blue) of each gene in each cluster were also observed. The characteristics of each CDR cluster were shown in Fig. 5, including TME features, treatment response, and gene expression profiles.

### Construction of CDRSig

**Figure 6:**
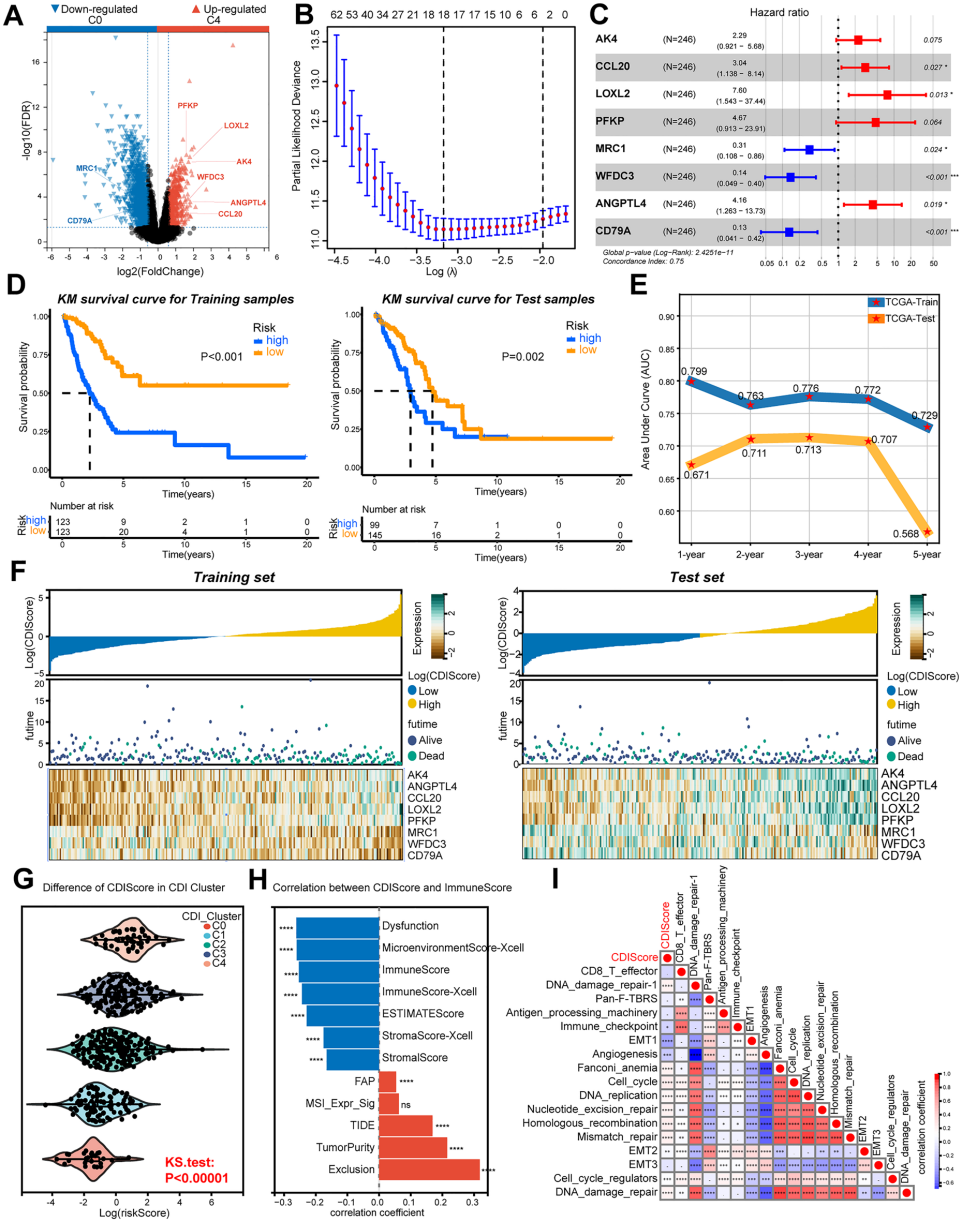
Construction of CDRSig. (**A**) Volcano plot for the up-regulated genes in CDR C4 and down-regulated genes in CDR C0. (**B**) LASSO COX regression analysis for DEGs in CDR C0 and C4. (**C**) Multivariate COX analysis of 8 genes in the CDRSig with the overall survival. (**D**) Survival curve for the training and test samples stratified by CDI. (**E**) Time-dependent ROC curve for TCGA training and test sets. (**F**) Overview of association among CDI, prognosis and 8 genes expression from CDRSig in training or test set. (**G**) Identification of CDI in CDR clusters. H, histogram of correlation between CDI and immune score. I, verification of RT/immune-related function analysis in CDI. (*, *P* < 0.05; **, *P* < 0.01; ***, *P* < 0.001; ****, *P* < 0.0001; ns, *P* > 0.05).

Building on the above results that the CDR C0 cluster was the more divergent, we compared these groups to identify DEGs. There were 171 downregulated genes and 35 upregulated genes in CDR C4 (|LogFC ≥ 1.5|, *p* < 0.05) (Fig. 6A). Univariate analysis and LASSO COX were used to identify prognostic and independent risk genes (Fig. 6B,C). Finally, 8 genes were selected to construct a cell death-related gene signature by multivariate COX regression analysis, including AK4, CCL20, LOXL2, PFKP, MRC1, WFDC3, ANGPTL4 and CD79A. Risk score of CDRSig (cell death index, CDI) was calculated: CDI = (− 1.187) × (expression level of MRC1) + (− 1.972) × (expression level of WFDC3) + (− 2.026) × (expression level of CD79A) + 0.827 × (expression level of AK4) + 1.112 × (expression level of CCL20) + 2.028 × (expression level of LOXL2) + 1.541 × (expression level of PFKP) + 1.426 × (expression level of ANGPTL4). CDI was able to differentiate cluster A and B in 4 cell death clusters (Supplementary Fig. 7A). In addition, the prognosis of the high-risk group was worse than the low-risk group in the train test, which was consistent with results in external test and validation sets (Fig. 6D, Supplementary Fig. 7B). Further receiver operating characteristic (ROC) curve illustrated the good prediction capability of CDRSig in 1 to 5 years survival possibility (Fig. 6E, Supplementary Fig. 7C). The correlation between CDI and genes expression in CDRSig was shown in Fig. 6F, as well as the prognosis prediction effect of CDRSig. Moreover, CDI could excellently discriminate CDR clusters (Fig. 6G). In order to verify the relevance among CDRSig, TME and RS, correlation was elucidated in Fig. 6H,I . We found that CDI was positively associated with FAP, TIDE, TumorPurity and TME exclusion, but negatively related with dysfunction, microenvironmentscore, immunescore, ESTIMATEscore and stromascore. Apart from the relevance of CDI in TME status, CDI was also implicated in DDR, cell cycle, DNA replication, nucleotide excision repair, homologous recombination, mismatch repair, and cell cycle regulators, all of which were relevant with RT-induced DDR mechanisms. Clinical characteristics analysis showed that gender, stage and RS were significantly associated with the high and low CDI groups (Supplementary Table 9). Data in Fig. 6 established the CDRSig to distinguish TME/DDR differentiation perfectly in various samples.

### Validation of CDIs for predicting ICIs and RT sensitivity in LUAD patients

**Figure 7:**
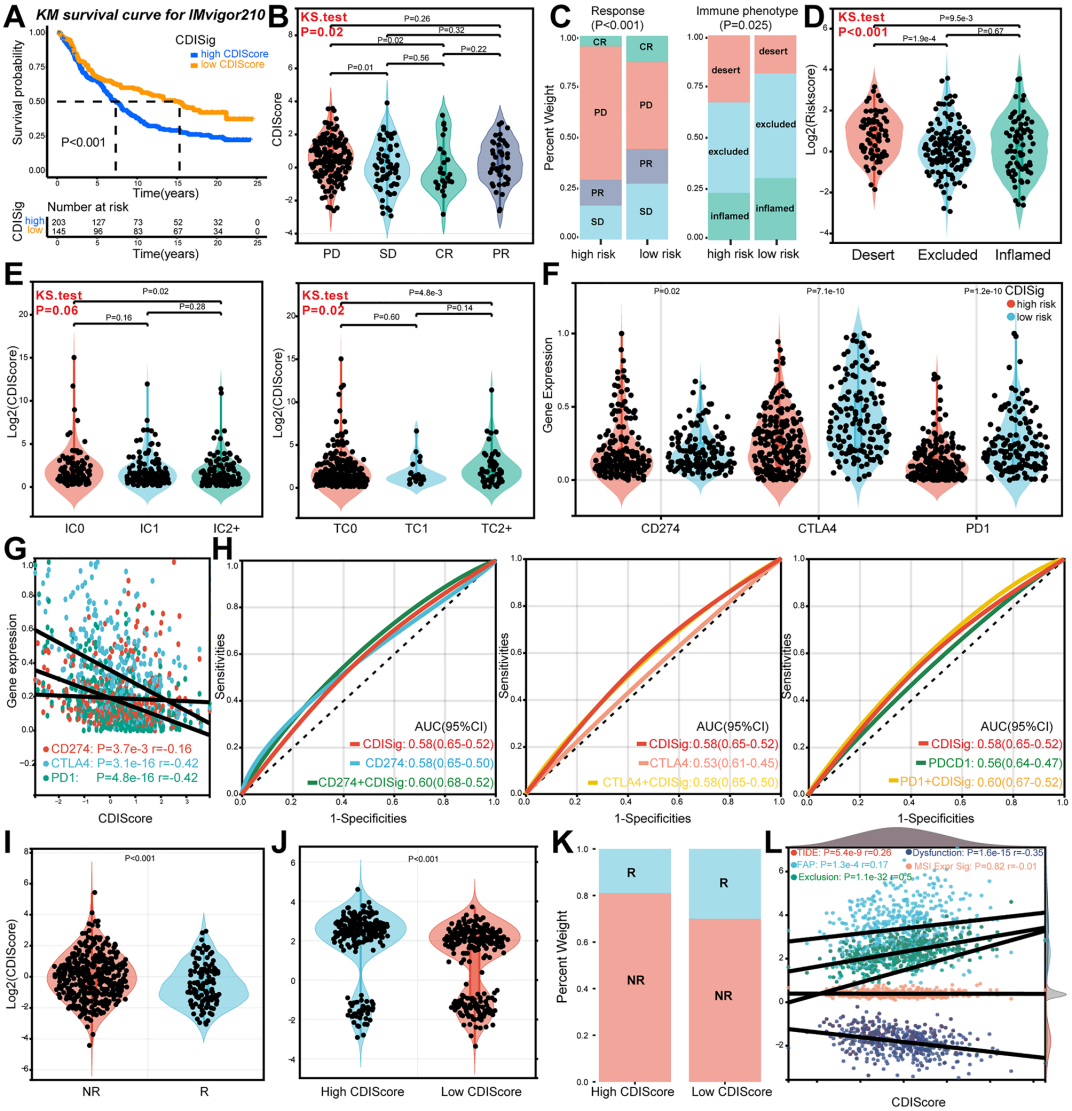
CDI predicts immunotherapy sensitivity of LUAD patients. (**A**) Survival curve for LUAD patients in IMvigor210 stratified by CDIScore. (**B**) Correlation between CDIscore and immunotherapy response using IMvigor210. PD, progressive disease. SD, stable disease. CR, complete response. PR, partial response. (**C**) Immunotherapy response and immune phenotype in high-risk CDIscore and low-risk CDIscore using IMvigor210. (**D**) Difference between CDIscore and immune phenotype (Kolmogorov–Smirnov test) using IMvigor210. (**E**) Correlation between CDIscore and PD-L1 expression in immune or tumor cells using IMvigor210 (Kolmogorov–Smirnov test). (**F**) Immune checkpoint molecule expression in the high- and low-risk groups using IMvigor210. (**G**) Continued variation between CDIscore and immune checkpoint molecule expression using IMvigor210. (**H**) AUC curve for survival stratified by CDRSig and CD274/CTLA4/PDCD1 using IMvigor210. (**I**) CDIscore difference for immunotherapy response using TIDE analysis. R, response. NR, non-response. (**J**) TIDE score in the low and high CDI groups. (**K**) Rate of immunotherapy response in low and high CDI groups. (**L**) Continued variation between CDIscore and immune phenotypes using TIDE analysis.

**Figure 8:**
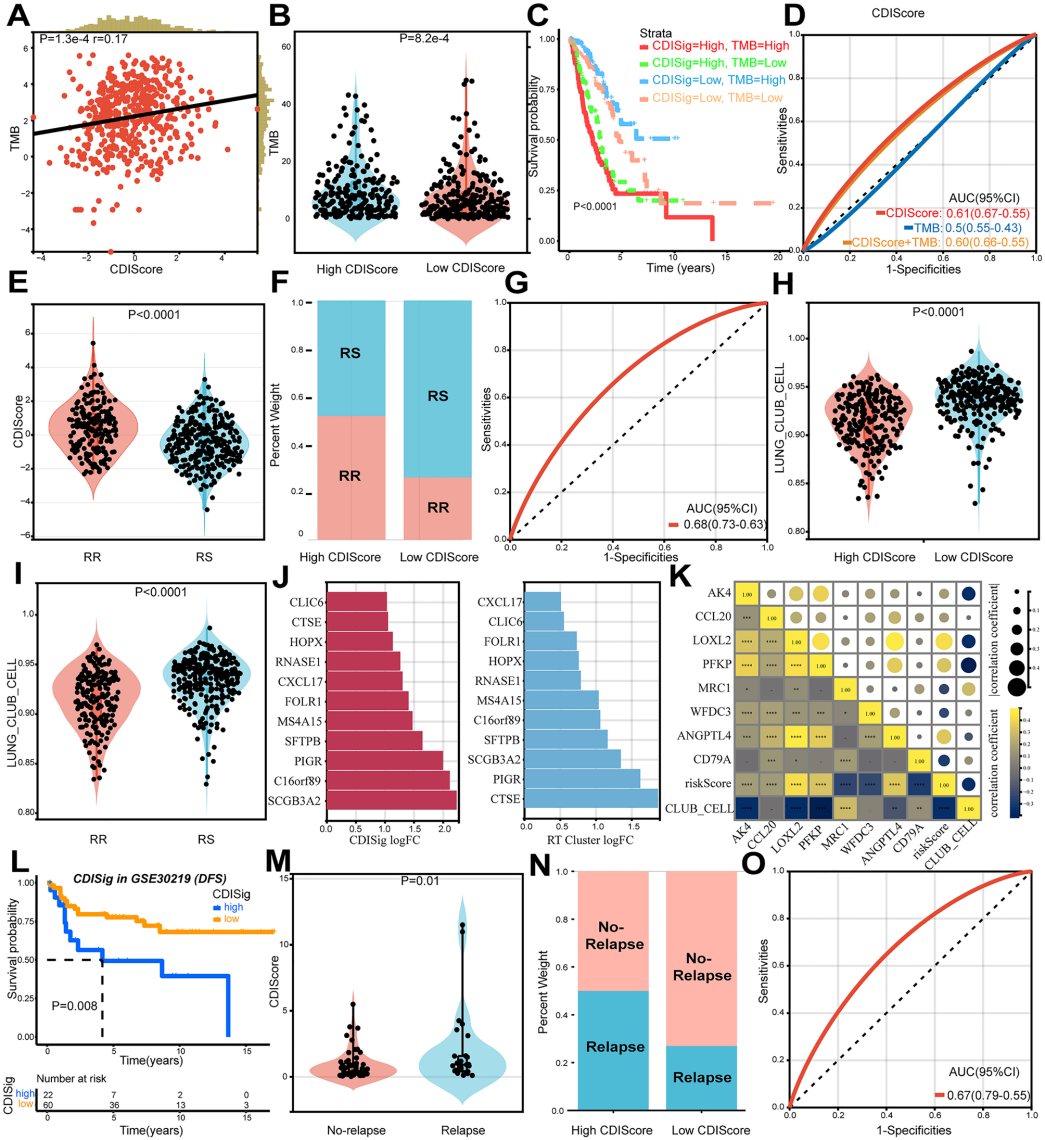
CDI predicts RS of LUAD patients. (**A**) Scatter diagram of association between tumor burden and CDI using TIDE analysis. (**B**) Tumor burden in the low and high CDI groups using TIDE analysis. (**C**) Survival analysis stratified by CDRSig and TMB using TIDE analysis. (**D**) AUC of CDIscore, tumor burden and combined CDIscore and tumor burden. (**E**) Difference of CDI between the RR and RS group. (**F**) Ratio of RR and RS in high and Low CDI. (**G**) ROC curve of CDIscore in RS prediction. (**H**) Percentage of lung club cells in high and low CDI. (**I**) Percentage of lung club cells in RR and RS. (**J**) Association of lung club cell-related gene expression between CDRSig and RT. (**K**) Correlation between lung club cells and gene expression in CDRSig. (**L**) Survival curve of CDRSig in GSE30219. (**M**) Correlation of CDI between tumor relapse and no-relapse. (**N**) Proportion of relapse in high and low CDI. (**O**) ROC curve of CDIScore in RS prediction.

Next, we tested the prediction ability of CDRSig to ICIs and RT responses using IMvigor210 data, TIDE web tool and GEO datasets (GSE78220, GSE100979, GSE115821). Kaplan–Meier (KM) survival curve uncovered that samples from IMvigor210 data (Fig. 7A) and GEO datasets (Supplementary Fig. 8A) with high CDI samples had shorter survival than low CDI ones. Figure 7B,C confirmed that patients in the low-risk group from IMvigor210 data in CDRSig were more likely to respond to immunotherapy, so as samples from GEO datasets (Supplementary Fig. 8B–D). Moreover, immune phenotypes were different between the high- and low-risk groups, with less immune cell infiltration in the high-risk and more in the low-risk groups (Fig. 7C,D). PD-L1 expression in immune and tumor cells had a significantly downward tendency with CDI increase (Fig. 7E). PD-L1 (CD274), PD-1 (PDCD1) and CTLA4 all had higher expression in the low-risk samples, suggesting the low-risk group with abundant ICI targets would be more likely to benefit from ICIs than the high risk one (Fig. 7F,G). In comparison with CDRSig or immune checkpoint alone, combined prediction would be enhanced (Fig. 7H). Using TIDE analysis, we found that the low CDI group had better prognosis and higher response rate receiving ICIs (Fig. 7I–K, Supplementary Fig. 8J). With the increase of CDI, there were upward trends among TIDE, FAP, tumor immune exclusion and TMB, while a downward in T cell dysfunction (Fig. 7L, Fig. 8A,B). By stratifying patients with CDI and TMB, we found that group with low CDI and high TMB had longer survival (Fig. 8C,D).

For RT responses, similar results were shown in Fig. 8E,F from the GSE30219 dataset. The high CDI group had worse prognosis following RT, and AUC curve showed the good prediction capability of CDRSig in the RS group (Fig. 8E–G). Moreover, there was more club cells in the low CDI samples, as well as more in the RS rather than RR group (Fig. 8H,I). Lung club cells were reported to regulate RT-inducing tumor cell killing, which in turn combined with ICIs to maximize anti-tumor functions^17^. Therefore, lung club cell-associated genes were discussed in CDRSig and RT clusters. The expression of secretoglobin family 3A member 2 (SCGB3A2), a characteristic gene of club cells^18^, was positively associated with the low-risk group, as well as the RS group (Fig. 8J). Genes in CDRSig were extremely correlated with CDI and lung club cells, again improving the significance of CDI in ICIs and RT prediction (Fig. 8K). Many patients after anti-tumor therapy had a temporary tumor regression then suffered from tumor relapse. Given the importance of tumor relapse, we sought the relationship between tumor relapse and CDRSig. The high CDI group had less disease-free survival time and would be more likely to have tumor recurrence (Fig. 8L–N). Furthermore, the AUC was 0.67 (Fig. 8O), indicating good predicative ability of CDRSig in tumor relapse. In conclusion, CDRSig might be a potential metric to measure ICI/RT responses and tumor recurrence.

### Silencing PFKP synergizes with RT to increase anti-tumor effect and activate cGAS/STING pathway

**Figure 9:**
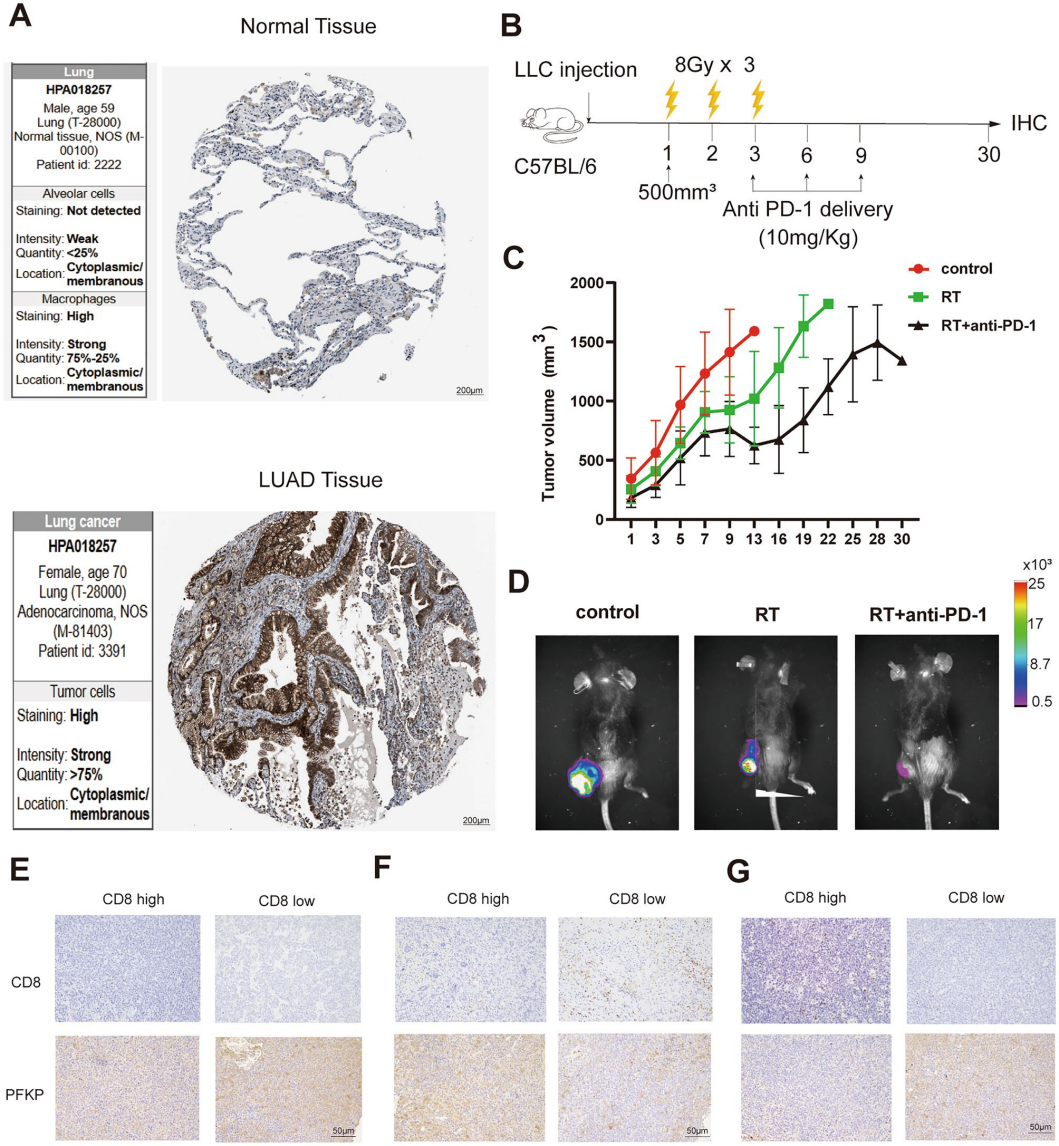
The role of PFKP in vivo. (**A**) Representative images of PFKP in clinical LUAD specimens from HPA website. (**B**) Schematic illustration of Lewis lung carcinoma model with RT or RT+ PD-1 combined treatment. (**C**) Survival of tumor-bearing mice. (**D**) Representative luciferase signal imaging of tumor-bearing mice. (**E**–**G**) IHC images for mice in control (**E**), RT (**F**) and RT+ PD-1 groups (**G**).

**Figure 10:**
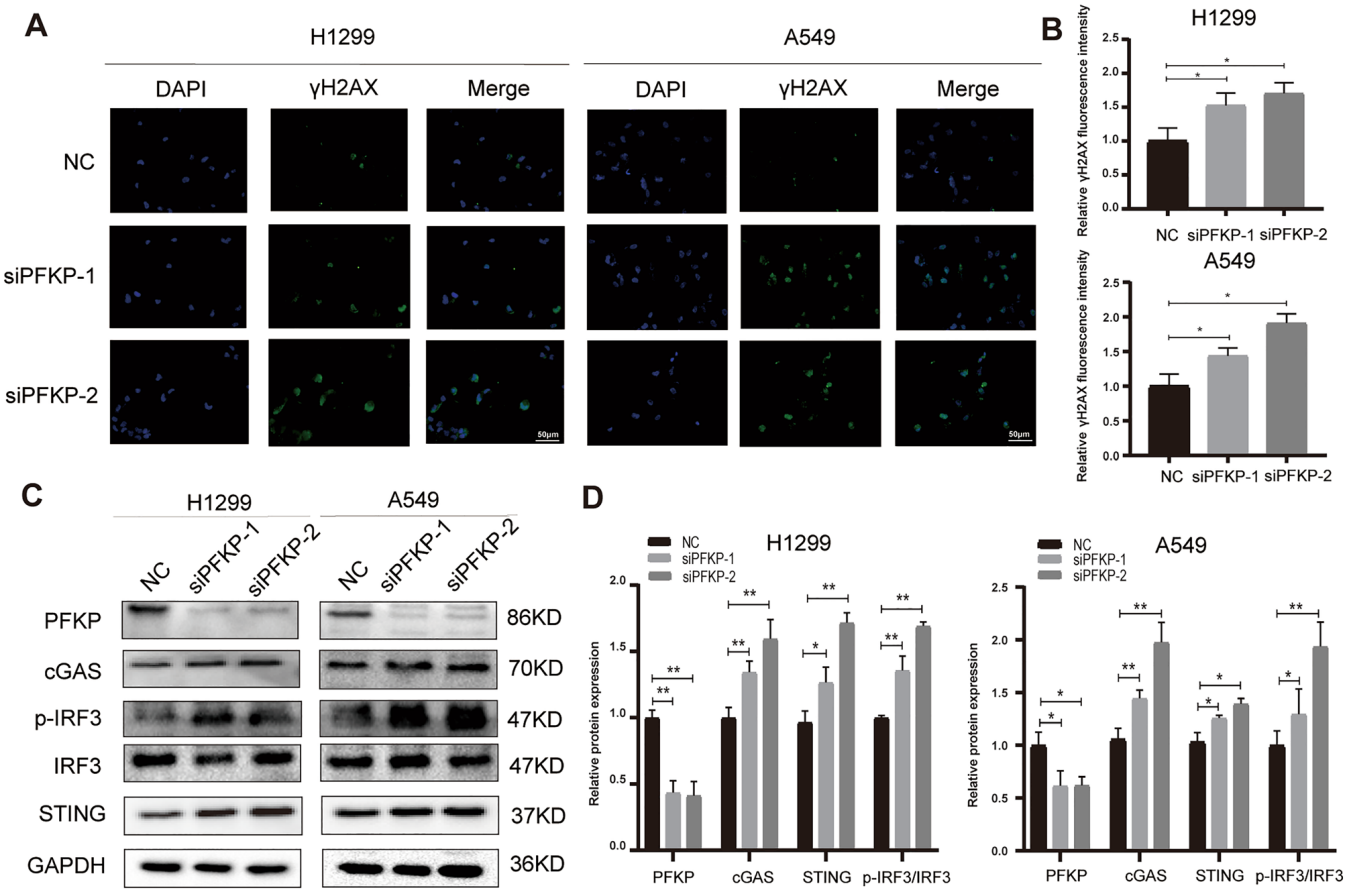
Inhibition of PFKP induces DNA damage and elevation of cGAS/STING pathway. (**A**) Immunofluorescence images of γH2AX. (**B**) The statistical analysis of γH2AX images. (**C**) Immunoblotting of cGAS/STING pathway with PFKP knockdown. (**D**) The statistical analysis of protein images. (*, *P* < 0.05; **, *P* < 0.01).

**Figure 11:**
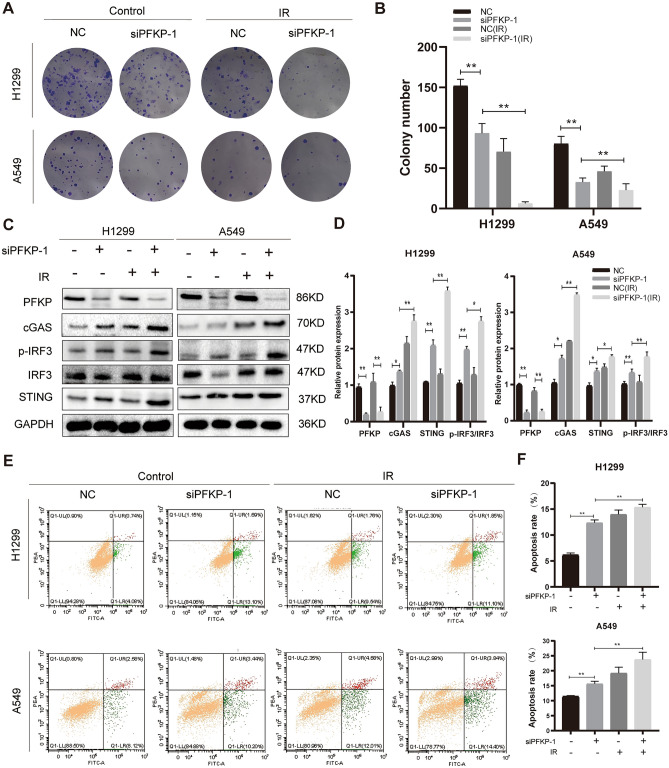
Inhibition of PFKP synergizes with RT to induce cytotoxicity of tumor cells and elevation of cGAS/STING pathway. (**A**) Synergism of RT and siPFKP on colony information. (**B**) The statistical analysis of colony information. (**C**) Immunoblotting of cGAS/STING pathway after PFKP knockdown and RT. (**D**) The statistical analysis of protein images. (**E**) Flow cytometry of cell apoptosis after PFKP knockdown and RT. (**F**) The statistical analysis of cell apoptosis. (*, *P* < 0.05; **, *P* < 0.01).

Phosphofructokinase platelet (PFKP) had high index in CDI. Using pathological image data from The Human Protein Atlas (HPA) pathology slide website (https://www.proteinatlas.org/), we found that the expression of PFKP was higher in LUAD tissues rather than normal regions (Fig. 9A). To further investigate the role of PFKP in TME, we constructed Lewis lung cancer model in C57BL/6 mice (LLC group, LLC group with radiation, LLC group with radiation and PD-1 antibodies) (Fig. 9B–D). Figure 9E–G showed that tissues with high expression of PFKP were immune-excluded or immune-desert types, which had less CD8+ T cell infiltration among the 3 groups. Cyclic GMP-AMP (cGAMP) synthase (cGAS) /stimulator of interferon genes (STING) pathway acts as intermediaries between IR-triggered DNA damage and interferon I-induced CD8+ T cell anti-tumor effects, mounting cytotoxity of IR and immunotherapy against tumors. PFKP knockdown in LUAD cells induced DNA damage and cGAS/STING pathway activation (Fig. 10A–D). Moreover, silencing PFKP collaborated with RT to restrain tumor cell survival (Fig. 11A,B), stimulated cGAS/STING signaling pathway (Fig. 11C,D) and increased cell apoptosis (Fig. 11E,F). Further GSEA for PFKP in LUAD also showed that PFKP was enriched in glycolysis, nucleotide excision repair, P53 signaling pathway, cell cycle and DNA replication (Supplementary Fig. 8H). We also performed protein–protein interaction analysis and found no relationship between PFKP and other genes in CDRSig (Supplementary Fig. 8I). These results evidenced the important predictive role of PFKP in TME and RT/immunotherapy responses.

## Discussion

Accumulating studies demonstrated that CDR patterns were vital for tumoricidal responses, but the complexity and variety of CDR molecules in TME were hard to be understood. In our study, we summarized 4 kinds of CDR genes from published researches. The genes with prognostic significance were used for subsequent consensus clustering analysis. Samples from TCGA database were divided into 5 categories by numbers of cell death events with poor prognosis (cluster A) in Upset plot. There were apparent differences among CDR clusters in clinical signatures, tumor immune microenvironment, immune cell infiltration, CSC characteristics and genome variation. Differences of TME, CSC characteristics and genome variation were reported to poor responses to ICIs and RT. Our data showed important associations among CDR clusters, TME and therapeutic sensitivity.

Cell death pathways consist of apoptosis, ferroptosis, pyroptosis and necroptosis. Considering various types of cells in TME, cell death including immune cells and tumor cells might cause different influence on anti-cancer immunity. On the one hand, DAMPs released from tumor cell death magnifies adaptive immunity response. On the other hand, chronic inflammation-induced by cell death might lead to immunosuppressive tumor microenvironment, which is full of myeloid-derived suppressor cells and impairs cancer therapy effectiveness^19^.In normal organism, apoptosis refers to a programmed cell death pattern for removing needless or aberrant cells^20^. This causes little strong immunity. Apoptosis mainly consists of extrinsic apoptosis-induced by tumor necrosis factor family and intrinsic apoptosis-mediated by mitochondria. Representative compounds have showed effectiveness in LUAD, such as Pyronaridine and LZ-101^21–23^. In TME, apoptosis process is a double-edged sword. Ferroptosis and apoptosis induced by Fascaplysin enhance the infiltration of CD4+ and CD8+ T cells, PD-L1 efficacy in mice^24^. Pancreatic progenitor cell differentiation and proliferation factor deficiency sensitizes NSCLC cells to NK cells killing by promoting apoptosis^25^. Auranofin induces apoptosis of NSCLC cells, which attracts natural killer cell recruitment and leads NK cell maturation^26^. However, blocking caspases evokes anti-tumor immunity combined with tumor shrinkage^27–29^, and caspase activity in apoptosis sometimes exerts immunosuppressive function in inhibiting type I interferon (IFN) response^30^ and suppressing DAMP activity^31^. Extrinsic apoptosis initiator like TNFa or TRAIL cause NSCLC cells secreting interleukin 8, leading to tumor progression and treatment resistance (chemotherapy, RT and ICIs)^32^. Other studies also reveal that NSCLC with poor prognosis often contain relatively high levels of constitutively apoptotic cells^33–35^. Resistance to apoptosis was a symbol for cancer characteristics, thereby how to utilize other CDR patterns in killing cancer cells have captured scientists’ attention. If apoptosis fails to induce cancer cell death, necroptosis would be the second choice for suppressing tumor^36^. RIP1, RIP3 and MLKL are the mediators of necroptosis. For example, Cryptotanshinone and SPA70 play important roles in improving anti-cancer effect as complementary options for apoptosis-resistant NSCLC by initiating necroptosis^37,38^. Yet, necroptosis also has pleiotropic functions in neoplastic progression, cytokines or DAMPs from which stimulate immune responses or increase the risk of developing tumors. High level of necrosis indicates good prognosis of NSCLC patients^39^. The release of interleukin (IL)-1α from necroptosis oncogenic cells generally promotes DCs secreting IL-12 to amplify anti-cancer immunity^40^, but sometimes necroptosis-derived IL-1α fosters tumor growth^41,42^. Multiple evidence supported that necroptosis-fostered inflammation recruit adaptive immune cells^43,44^, others indicated that ameliorating necroptosis would turn TME from immunosuppressive to immune-activated status (decreasing myeloid-derived suppressor cell, tumor-associated macrophage and increasing T cell infiltration)^45,46^. Pyroptosis is characterized by inflammasome, accompanying with cell swelling, cell membrane lysis. As a result, the release of intracellular content triggers inflammation. Through stimulating NLRP3/caspase-1/ Gasdermin (GSDM) axis, Simvastatin and NO.0449-0415 are recognized as pyroptosis compounds in NSCLC cells^47,48^. In TME, pyroptosis exerts anti-tumor function via inhibiting cell survival and shows pro-cancer effects via reprogramming cancer-suitable microenvironment^49^. Activated Caspase 4/11 of T cells would lead to GSDM cleavage, mainly increasing CD8 effector T cells proportion by upregulating Ovalbumin (OVA)^50^, while the expression of GSDMD showed a positive correlation with aggressive features (tumor volume, tumor stage) in NSCLC^51^. Ferroptosis, a form of CDR modalities, works by accumulating lipid peroxides to induce cell death. Representative drugs, like Curcumin and Ammonium ferric citrate, are treatment-induced ferroptosis in NSCLC^52–54^. IFNy-secreted from CD8+ T cells would finally cause tumor cell ferroptosis, and such phenomenon also has been observed in tumor-bearing mice after PD-L1 deprivation^55^. CD8 T cells activated by ICIs stimulate ferroptosis in turn, amplifying immune response in positive feedback loops^55^. CD36 of CD8 T cells-induced ferroptosis impairs cytokine (IFNG, GZMB) production and decreases anti-tumor immunity in metastatic lung tumor^56^. Moreover, iron-mediated cell death has been used in clinical anti-cancer strategies, while excessive iron accumulation might lead to tumor development acceleration^57,58^. Undoubtedly, there is a highly-close crosstalk among immune response and cell death patterns, but the contradictory functions would hinder the wide use of cell death in targeting cancers treatments. To this end, figuring out the accurate mechanism in suppressing tumor growth and promoting cancer progression are vital for modulating CDR therapy effect.

In this work, we innovatively put forward a CDR score formula, CDRSig, to identify CDR genes in LUAD patients. CDRSig was significantly associated with LUAD prognosis, clinical stage, immune cell (especially CD8+ T cell) infiltration, anti-tumor immune cell-related molecule levels and ICI responses. ICI sensitivity depends on multiple factors, including the presence of CD8+ T cells, PD-L1 expression, immune cell infiltration in TME. Considering scarce immune cells/molecules in CDR C4 group, FAP (related with intratumoral CD8+ T cells deficiency) might be one of the potential mechanisms for unsatisfactory prognosis^15^. Moreover, recent study found that T cells function rather than T cell content in immune-infiltrated LUAD might be the indicator for ICI effectiveness. There was a subset of antigen-activated T cell aberrant in gene expression, thereby leading to ICI resistance in immune-infiltrated LUAD patients. It was the downregulation of TIM3-coding gene (HAVCR2) partly determining ICI response that contributed to the failure of activated T cells transforming to effector T cells initiated by ICIs, which could be restored by addition of IL-2/IL-12 ^14^. Notably, CDR C0 group was infiltrated with co-inhibitors and had higher expression of HAVCR2 than C2, C3, C4, providing sites for ICIs to combine and increasing anti-cancer CD8+ T cells immunity. Adopting IL-2/IL-12 might be an effective way for C2, C3, C4 group to reactivate T effector cell function and enhance ICI response rate.

Combined approach of RT and ICIs represents promising clinical outcomes in remodeling TME and causing DNA damage in tumor cells. For instance, cGAS-STING pathway plays vital role in sensing cytosolic DNA and stimulating innate immune response (type I IFNs), giving interesting prospect for synergy between DNA damage-related therapies (like RT) and immunotherapy in tumor^59^. Data in our studies showed the negative association between CDRSig and cGAS-STING, suggesting the reliability of CDRSig in predicting response for LUAD patients with combination of RT and ICIs (Supplementary Fig. 8E,F). Cell-to-cell contact is essential for tumor-infiltration lymphocytes to eradicate malignant tumor cells. So, the exclusion of immune cells in some tumor stroma (“cold” tumor) blunted anti-tumor immunity, altering which would significantly unleash ICI function. RT reprograms TME in promoting polarization of M2 to M1 macrophages, increasing MHC-I expression and ICD or enabling T cell adhesion to tumor cells by upregulating cell-contact molecules levels and T cell-trafficking chemokines^60^. Therefore, with the increase of tumor antigens release and alternation in immune-exclusion TME after RT and ICIs, better prognosis might be shown in CDR C1-C4 groups. Recent studies revealed that RT could specifically activate lung club cells, releasing proteins conducive to immunotherapy. In addition, scarcity of oxygen would also cause radiation resistance and disrupt immune cells function in TME. After using oxidative phosphorylation (OXPHOS) inhibitors, the increase of RT/ICI sensitivity and combination of RT&ICI response with ICD improvement were observed^61^. Consistently, C0 group had more lung club cells infiltration and better response to ICIs/RT than C1, C2, C3 and C4, while OXPHOS function would be enriched in C4 with higher RS possibility. Thus, for CDR C1, C2, C3, C4 groups who were resistant to synergized RT and ICIs or intolerant of RT, we provided some targeted suggestion like oxidative phosphorylation inhibitors administration, using lung club cells-specific proteins (CC10) or IL-2/IL-12 (restore ICI response in T cells for HAVCR2 deficiency) except synergistic RT and ICI therapies^17^.

Pathological data from TCGA patients and mouse models showed that CDI score was notably linked to TME subtypes. Specially, samples with high CDI scores had immune excluded TME, while low CDI tended to obtain immune inflamed TME. Genes in CDR signature had been reported to participate in tumor immunity process^62–70^, in which LOXL2 and PFKP were apparently with high index in CDRSig. LOXL2, one of lysyl oxidase gene family, participates in malignant tumor progression and extracellular collagen deposition^71–75^. For cancer treatment, ablation LOXL2 restores cancer cells RS, increases intratumoral CD8+ T cell infiltration, declines exhausted CD8+ T cells, thus reducing tumor burden and resensitizing anti-PD-L1 resistant lung cancer cells^76,77^. PFKP, targeted by hypoxia-inducible factor 1 alpha (HIF1a), has close relation with tumor aerobic glycolysis, tumor growth, immune evasion and reducing metabolic load under glucose starvation^78–81^. High expression of PFKP declines sensitivity to cisplatin in NSCLC cells via upregulating NFkB signaling pathway^82^. PFKP and ANGPTL4 are predicted as hypoxia-related genes in LUAD^83^. Moreover, DNA damage repair molecule Ataxia-Telangiectasia mutated kinase mediates energy metabolism reprogram by HIF1a/PFKP axis^84^. As no literature showed direct relation among PFKP, tumor immune environment and RS, we further validated its function in vivo and in vitro. The tumors with high PFKP levels had immune deficient TME. In addition, PFKP deficiency in LUAD cells triggered genome instability, thus inducing radiation sensitivity and cGAS/STING pathway activation. These results indicate that PFKP from CDRSig might have promising effects in regulating immunotherapy and radiation response, revalidating excellent predictability of CDRSig.

There were some limitations in our study, relying on retrospective data and lacking enough clinical LUAD samples to verify CDI. Our laboratory would collect more clinical LUAD specimens with detailed treatment information to validate the predictive ability of CDI on radiation responses in our further analysis. Our future research will test cell subgroup heterogeneity among CDR clusters in single-cell data, identifying specific cell group-derived certain gene as a therapeutic target of non-small cell lung cancer. We will also try to develop a machine learning prediction model to improve the AUC.

Our studies creatively proposed a comprehensive CDRSig via summarizing the relationship between cell death-related genes and patient prognosis upon RT or immunotherapy. This CDRSig could effectively predict patients’ prognosis and therapy responses, and also stratify patients and provide personalized treatment alternations.

## Materials and methods

### Data collection and process

The workflow of this study is displayed in Supplementary Fig. 1. RNA-sequencing data (FPKM normalized) and clinical information were obtained from The Cancer Genome Atlas (TCGA; https://portal.gdc.cancer.gov). After excluding patients with survival less than 30 days, 490 LUAD patients were included, of which 57 patients received RT. Two external test sets (GSE30219 and GSE13213) and 1 external validation set (Merge set of GSE19188, GSE31210 and GSE50081) were collected from the Gene Expression Omnibus (GEO; http://www.ncbi.nlm.nih.gov/geo). Among the 2 external test sets, GSE30219 existed the completed tumor relapse annotation and disease-free survival time (DFS). Moreover, GSE78220, GSE100797 and GSE115821 were integrated into the immunotherapy data set to predict the immunotherapy responses. GSE126044 was used for predicting immune therapy response in non-small cell lung cancer patients receiving anti PD-1 immunotherapy. All the raw CEL files downloaded from GEO were treated using the Robust Multichip Average algorithm for background adjustment and quantile normalization. The detailed information is recorded in Supplementary Table 10.

### Construction of CDR cluster

We obtained the marker genes for apoptosis, necrosis^85^ and pyroptosis^86^ from precious publications. Ferroptosis markers were downloaded from FerrDb database (http://www.datjar.com:40013/bt2104)^87^. Based on different cell death markers significantly correlated with overall survival, 490 LUAD patients were divided into 2 groups using consensus clustering (k = 2) with a subsampling ratio of 0.9 and a total of 1,000 permutation tests. By comparing the difference of overall survival between the 2 groups, LUAD patients were classified into apoptosis/ferroptosis/necrosis/pyroptosis groups with favorable or poor prognosis. Finally, according to the number of cell death events with poor survival, CDR cluster was successfully constructed (C0:0 events of unfavorable prognosis, C1:1 events of of unfavorable prognosis, C2:2 events of unfavorable prognosis, C3:3 events of unfavorable prognosis, C4:4 events of unfavorable prognosis).

### Calculation of immunological characteristics scores in LUAD

We accessed the immunescore, stromalscore, microenvironmentscore and tumorpurity of LUAD patients using Xcell and Estimation of Stromal and Immune cells in Malignant Tumour tissues using Expression data (ESTIMATE) algorithms 22,23. Gene data for 29 immune cells were obtained from TCGA database and evaluated by the single sample gene set enrichment analysis (ssGSEA) algorithm to measure the overall levels of immune cells. In addition, Xcell, MCPcounter, Cibersort and CibersortX algorithms were used to quantify the abundance of CD8+ T cells. Likewise, we calculated the enrichment scores of lung parenchymal and non-parenchymal cells using the ssGSEA algorithm based on cell type signature gene sets from Molecular Signatures Database (MsigDB, http://www.gsea-msigdb.org/gsea/msigdb). Moreover, 122 immunomodulators, such as chemokines, MHC, receptors and immunostimulants were collected from previous studies 24,25. Tumor immune circulation is a complex process consisted of 7 steps 26: (1) cancer antigen release, (2) cancer antigen presentation, (3) priming and activation, (4) trafficking of T cells to tumors, (5) infiltration of T cells into tumor, (6) recognition of cancer cells by T cells, (7) killing of cancer cells. We quantified the importance of every steps according to ssGSEA algorithm based on the markers for each step downloaded from the tracking tumor immune phenotype website (http://biocc.hrbmu.edu.cn/TIP). Tumor immune dysfunction and exclusion (TIDE) scores and MSI expression were estimated with the TIDE website tools (http://tide.dfci.harvard.edu) 27. It has been reported that TIDE could accurately predict the immunotherapy responses of lung cancer patients.

### Assessment of tumor stemness and well-known tumor signals or pathways

Cancer stem cells (CSCs) have been indicated to promote tumor initiation and recurrence. According to Maciej Wiznerowicz et al. 28, tumor stemness indexes such as mRNAsi (tumor stemness index based on mRNA expression), mDNAsi (tumor stemness index based on mDNA expression), DMPsi (differentially methylated probes-based stemness index), EREG-mDNAsi (tumor stemness index based on stem cell epigenetic regulation-related genes) were generated. In addition, 37 cancer stem surface markers were collected from Won-Tae Kim et al. 29. Xiao et al. summarized the gene signatures of 10 important carcinogen signals such as Wnt, RAS, PI3K, NOTCH, MYC, Hippo, cell cycle, TGF-beta, TP53 and NRF2 25. The immune-related genes such as immune checkpoint, antigen processing machinery, CD8 T-effector and epithelial-mesenchymal transition were gathered. “ssGSEA” method was performed to achieve the enrichment scores for each characteristic.

### Gene mutation and CNV analysis

Somatic variants with the Mutation Annotation Format for LUAD patients were acquired from the TCGA database. The waterfall chart of gene mutation and CNV was illustrated with the ‘maftools’ R package. According to the following formula: (mutation frequency with a number of variants)/the length of exons (38 million), we calculated the TMB scores for LUAD patients. GISTIC.2 on the Genepattern website (https://cloud.genepattern.org) was performed to analyze amplified genomes and missing gene sequences (Reference set: human hg38 genome sequence) 30.

### Identification of radiosensitive (RS) and radioresistant (RR) groups

31 RS signatures were identified by Han Sang Kim et al. using integrative metanalysis 31. Based on the 14 RS signatures which had significant correlation with overall survival, the 490 LUAD patients were classified into 2 groups using consensus cluster analysis. The overall survival of RT patients in both groups was compared. The cluster with better prognosis was identified as RS group, while the other as RR group.

### Construction of cell death risk signature (CDRSig)

In the 5 CDR cluster patterns, the difference between C0 and C4 was the most significant, which could become the typical of CDR cluster. Thus, the differential genes (DEGs) between C1 and C4 (|LogFC ≥ 1.5|, *p* < 0.01) were selected to construct the gene signature. Univariate Cox regression analysis was chosen to screen out the prognosis-related DEGs. Least absolute shrinkage and selection operator (LASSO) was performed to find more vital DEGs by generating a penalty function to compress the variable coefficients in the regression model. After multivariate COX regression analysis, CDRSig was successfully constructed. The expression values of the selected DEGs weighted by the multivariate COX regression coefficient were converted to the risk score. The “survival” and “survivalROC” R packages were used to illustrated Kaplan–Meier curves and time-dependent receiver operating characteristic (ROC) curve.

### Cells and RNA interference

Humanfi NSCLC cell lines (H1299 and A549) were cultured in RPMI-1640 (Hyclone, USA), which were purchased from Type Culture Collection of the Chinese Academy of Sciences (Shanghai, China). PFKP was knockdown with small interfering RNAs (siRNAs) using lipofectamine transfection reagent (Invitrogen). siRNA sequences were presented in Supplementary Table 11.

### Immunofluorescence

Cells on coverslips were fixed with paraformaldehyde (4%, room temperature, 30 min) and permeabilized with Triton X-100 (0.5%, room temperature, 15 min). They were then blocked in bovine serum albumin (5%, room temperature, 1 h) and incubated with γH2AX antibody (4 °C, overnight). After incubated with secondary antibodies, cells were stained with DAPI for 15 min and images were taken under fluorescent microscope.

### Immunoblotting

Non-irradiated (control) cells or cells irradiated (IR) with 10 Gy were collected and proteins were extracted by RIPA cell lysis buffer (Beyotime, China) and centrifugated (12,000 rpm, 4 °C, 10 min). Supernatants were boiled with 5 X SDS loading buffer (100 °C, 10 min). After separated by 10% SDS-PAGE gels, proteins were transferred onto PVDF membranes. We blocked membranes with 5% non-fat milk and then incubated with primary antibodies (4 °C, overnight). After washing, membranes were incubated secondary antibodies for 1 h. We detected proteins with Bio-Rad Image Lab software. Primary antibodies were presented in Supplementary Table 12.

### Colony formation assay and flow cytometry

For colony formation assay, cells after transfection (1 × 10^3^ cells/well) and irradiated with 6 Gy were seeded into 6-well plates for 2 weeks. For apoptosis assay, single-cell suspensions after transfection and irradiated with 10 Gy were incubated with Annexin V-FITC staining solution at 4 °C for 15 min and then incubated with propidium iodide (PI) solution for 5 min. CytoFLEX system was used to analyze apoptosis assays.

### Mice

C57BL/6 mice (6 weeks) were obtained from the Vital River Laboratories (Beijing, China). Lewis cells (1 × 10^7^ cells/100 μl) were injected subcutaneously into the right armpits. When tumor volume reached 500 mm3 (reckoned as Day 1), mice were divided into 3 groups: control group (no treatment), RT group (8 Gy × 3), RT+ anti PD-1 group (8 Gy × 3 + 10 mg/kg anti-PD-1). Tumor regions were irradiated by the small animal radiation research platform (PXI X-RAD 225Cx, Gulmay, CT, USA) with 8 Gy/day for 3 days. PD-1 antibody (Bioxcell) was administered (i.p.) at 10 mg/kg/day for 3 times (Day 5, 8, 13). When tumor volume reached 2000 mm3 or tumor had developed for 30 days, mice were sacrificed using intraperitoneal injection of 150–200 mg/kg of sodium pentobarbital for tumor dissection. Animal studies were performed in compliance with institutional guidelines and approved by Institutional Review Board at Zhongnan Hospital of Wuhan University, protocol numbers (ZN2021087). All experiments were performed according to ARRIVE guidelines.

### Statistical analysis

We used Pearson and Spearman correlation coefficients to determine the correlation between variables. The unpaired student’s t-test was used to estimate the statistical significance of normally distributed variables, and the Mann–Whitney U test (also known as Wilcoxon rank sum test) was used to analyze non-normally distributed variables. To compare 2 or more groups, Kruskal–Wallis and one-way ANOVA tests were used. The two-sided Fisher’s exact test analyzes the contingency table. The “Surv-cut point” function in the R package ‘Survminer’ was used to evaluate the critical value of each data set. The Kaplan–Meier method was used to generate survival curves for the subgroups of each data set, and the log-rank (Mantel–Cox) test was used to determine statistically significant differences. A univariate Cox proportional hazard regression model was used to calculate the hazard ratio. All statistical analyses used R (https://www.r-project.org/), with a *P* value < 0.05 (two-tailed) indicating significant differences.

### Ethics statement

All animal studies were approved by Institutional Review Board at Zhongnan Hospital of Wuhan University, protocol numbers (ZN2021087 approved on 22 July 2021). The patients’ data from public database TCGA and GEO have obtained ethical approval.

### Supplementary Information

Supplementary Figure 1.

Supplementary Figure 2.

Supplementary Tables.

## Data Availability

The datasets analyzed for this study can be found in the Gene Expression Omnibus (https://www.ncbi.nlm.nih.gov/geo/) and TCGA database. GEO datasets and TCGA LUAD data used for this study can be acquired from online database (https://www.ncbi.nlm.nih.gov/geo/query/acc.cgi) (http://cancergenome.nih.gov). Other original contributions presented in the study are included in the article/Supplementary Material. Any inquiry should be submitted to the corresponding authors.

## References

[1] Han L (2020). Gene signature based on B cell predicts clinical outcome of radiotherapy and immunotherapy for patients with lung adenocarcinoma. Cancer Med..

[2] Tokito T (2016). Predictive relevance of PD-L1 expression combined with CD8+ TIL density in stage III non-small cell lung cancer patients receiving concurrent chemoradiotherapy. Eur J Cancer..

[3] Dovedi SJ (2014). Acquired resistance to fractionated radiotherapy can be overcome by concurrent PD-L1 blockade. Cancer Res..

[4] Wei J (2021). Sequence of alphaPD-1 relative to local tumor irradiation determines the induction of abscopal antitumor immune responses. Sci. Immunol..

[5] Theelen W (2019). Effect of pembrolizumab after stereotactic body radiotherapy vs pembrolizumab Alone on tumor response in patients with advanced non-small cell lung cancer: Results of the PEMBRO-RT phase 2 randomized clinical trial. JAMA Oncol..

[6] Elbanna M (2021). Clinical and preclinical outcomes of combining targeted therapy with radiotherapy. Front. Oncol..

[7] Ahmed A, Tait SWG (2020). Targeting immunogenic cell death in cancer. Mol. Oncol..

[8] Garg AD, Agostinis P (2017). Cell death and immunity in cancer: From danger signals to mimicry of pathogen defense responses. Immunol. Rev..

[9] Ichim G, Tait SW (2016). A fate worse than death: apoptosis as an oncogenic process. Nat Rev Cancer..

[10] Brandt WS (2018). Factors associated with distant recurrence following R0 lobectomy for pN0 lung adenocarcinoma. J. Thorac. Cardiovasc. Surg..

[11] Milas L (2005). Targeting molecular determinants of tumor chemo-radioresistance. Semin. Oncol..

[12] Chouaid C (2018). Adjuvant treatment patterns and outcomes in patients with stage IB-IIIA non-small cell lung cancer in France, Germany, and the United Kingdom based on the LuCaBIS burden of illness study. Lung Cancer..

[13] Nagarsheth N (2017). Chemokines in the cancer microenvironment and their relevance in cancer immunotherapy. Nat. Rev. Immunol..

[14] Horton BL (2021). Lack of CD8(+) T cell effector differentiation during priming mediates checkpoint blockade resistance in non-small cell lung cancer. Sci. Immunol..

[15] Desbois M (2020). Integrated digital pathology and transcriptome analysis identifies molecular mediators of T-cell exclusion in ovarian cancer. Nat. Commun..

[16] Ogawa Y (2021). Three distinct stroma types in human pancreatic cancer identified by image analysis of fibroblast subpopulations and collagen. Clin. Cancer Res..

[17] Ban Y (2021). Radiation-activated secretory proteins of Scgb1a1 (+) club cells increase the efficacy of immune checkpoint blockade in lung cancer. Nat Cancer..

[18] Kotton DN, Morrisey EE (2014). Lung regeneration: mechanisms, applications and emerging stem cell populations. Nat Med..

[19] Strilic B (2016). Tumour-cell-induced endothelial cell necroptosis via death receptor 6 promotes metastasis. Nature.

[20] Czabotar PE (2014). Control of apoptosis by the BCL-2 protein family: implications for physiology and therapy. Nat. Rev. Mol. Cell Biol..

[21] Derakhshan A (2017). Therapeutic small molecules target inhibitor of apoptosis proteins in cancers with deregulation of extrinsic and intrinsic cell death pathways. Clin. Cancer Res..

[22] Rousalova I (2013). Minnelide: a novel therapeutic that promotes apoptosis in non-small cell lung carcinoma in vivo. PLoS One.

[23] Li S (2024). Targeted therapy for non-small-cell lung cancer: New insights into regulated cell death combined with immunotherapy. Immunol. Rev..

[24] Luo L, Xu G (2022). Fascaplysin induces apoptosis and ferroptosis, and enhances anti-PD-1 immunotherapy in non-small cell lung cancer (NSCLC) by promoting PD-L1 expression. Int. J. Mol. Sci..

[25] Zheng QW (2022). PPDPF promotes lung adenocarcinoma progression via inhibiting apoptosis and NK cell-mediated cytotoxicity through STAT3. Oncogene.

[26] Freire Boullosa L (2021). Auranofin reveals therapeutic anticancer potential by triggering distinct molecular cell death mechanisms and innate immunity in mutant p53 non-small cell lung cancer. Redox Biol..

[27] Giampazolias E (2017). Mitochondrial permeabilization engages NF-kappaB-dependent anti-tumour activity under caspase deficiency. Nat. Cell Biol..

[28] Rongvaux A (2014). Apoptotic caspases prevent the induction of type I interferons by mitochondrial DNA. Cell.

[29] White MJ (2014). Apoptotic caspases suppress mtDNA-induced STING-mediated type I IFN production. Cell.

[30] Ning X (2019). Apoptotic caspases suppress type i interferon production via the cleavage of cGAS, MAVS, and IRF3. Mol. Cell..

[31] Kazama H (2008). Induction of immunological tolerance by apoptotic cells requires caspase-dependent oxidation of high-mobility group box-1 protein. Immunity.

[32] Favaro F (2022). TRAIL receptors promote constitutive and inducible IL-8 secretion in non-small cell lung carcinoma. Cell Death Dis..

[33] Morana O (2022). The apoptosis paradox in cancer. Int. J. Mol. Sci..

[34] Mangili F (1998). Cell loss and proliferation in non-small cell lung carcinoma: correlation with histological subtype. Eur. J. Histochem..

[35] Tormanen U (1995). Enhanced apoptosis predicts shortened survival in non-small cell lung carcinoma. Cancer Res..

[36] Gong Y (2019). The role of necroptosis in cancer biology and therapy. Mol Cancer..

[37] Singh AP (2019). Targeted therapy in chronic diseases using nanomaterial-based drug delivery vehicles. Signal Transduct. Target. Ther..

[38] Wu M (2013). Deoxypodophyllotoxin triggers necroptosis in human non-small cell lung cancer NCI-H460 cells. Biomed. Pharmacother..

[39] Lim JH (2021). Low-level expression of necroptosis factors indicates a poor prognosis of the squamous cell carcinoma subtype of non-small-cell lung cancer. Transl. Lung Cancer Res..

[40] Schmidt SV (2015). RIPK3 expression in cervical cancer cells is required for PolyIC-induced necroptosis, IL-1alpha release, and efficient paracrine dendritic cell activation. Oncotarget.

[41] Hanahan D, Weinberg RA (2011). Hallmarks of cancer: The next generation. Cell.

[42] Greten FR, Grivennikov SI (2019). Inflammation and cancer: triggers, mechanisms, and consequences. Immunity.

[43] Kaczmarek A (2013). Necroptosis: the release of damage-associated molecular patterns and its physiological relevance. Immunity.

[44] Biswas SK, Mantovani A (2010). Macrophage plasticity and interaction with lymphocyte subsets: cancer as a paradigm. Nat. Immunol..

[45] Ostrand-Rosenberg S, Sinha P (2009). Myeloid-derived suppressor cells: Linking inflammation and cancer. J Immunol..

[46] Seifert L (2016). The necrosome promotes pancreatic oncogenesis via CXCL1 and mincle-induced immune suppression. Nature.

[47] Long K (2021). Small-molecule inhibition of APE1 induces apoptosis, pyroptosis, and necroptosis in non-small cell lung cancer. Cell Death Dis..

[48] Wang F (2018). Simvastatin suppresses proliferation and migration in non-small cell lung cancer via pyroptosis. Int. J. Biol. Sci..

[49] Xia X (2019). The role of pyroptosis in cancer: pro-cancer or pro-"host"?. Cell Death Dis..

[50] Xi G (2019). GSDMD is required for effector CD8(+) T cell responses to lung cancer cells. Int. Immunopharmacol..

[51] Gao J (2018). Downregulation of GSDMD attenuates tumor proliferation via the intrinsic mitochondrial apoptotic pathway and inhibition of EGFR/Akt signaling and predicts a good prognosis in non-small cell lung cancer. Oncol. Rep..

[52] Zhang Q (2019). Curcumin potentiates the galbanic acid-induced anti-tumor effect in non-small cell lung cancer cells through inhibiting Akt/mTOR signaling pathway. Life Sci..

[53] Zhang L (2019). Identification of compound CA-5f as a novel late-stage autophagy inhibitor with potent anti-tumor effect against non-small cell lung cancer. Autophagy.

[54] Wu W (2021). Ammonium ferric citrate induced ferroptosis in non-small-cell lung carcinoma through the inhibition of GPX4-GSS/GSR-GGT axis activity. Int. J. Med. Sci..

[55] Wang W (2019). CD8(+) T cells regulate tumour ferroptosis during cancer immunotherapy. Nature.

[56] Ma X (2021). CD36-mediated ferroptosis dampens intratumoral CD8(+) T cell effector function and impairs their antitumor ability. Cell Metab..

[57] Torti SV, Torti FM (2013). Iron and cancer: more ore to be mined. Nat. Rev. Cancer..

[58] Crielaard BJ (2017). Targeting iron metabolism in drug discovery and delivery. Nat. Rev. Drug Discov..

[59] Kwon J, Bakhoum SF (2020). The cytosolic DNA-sensing cGAS-STING pathway in cancer. Cancer Discov..

[60] Menon H (2019). Role of radiation therapy in modulation of the tumor stroma and microenvironment. Front. Immunol..

[61] Boreel DF (2021). Targeting oxidative phosphorylation to increase the efficacy of radio- and immune-combination therapy. Clin. Cancer Res..

[62] Jan YH (2019). Adenylate kinase 4 modulates oxidative stress and stabilizes HIF-1alpha to drive lung adenocarcinoma metastasis. J. Hematol. Oncol..

[63] Jan YH (2012). Adenylate kinase-4 is a marker of poor clinical outcomes that promotes metastasis of lung cancer by downregulating the transcription factor ATF3. Cancer Res..

[64] Fan T (2022). CCL20 promotes lung adenocarcinoma progression by driving epithelial-mesenchymal transition. Int. J. Biol. Sci..

[65] Xia L (2022). RORgammat agonist enhances anti-PD-1 therapy by promoting monocyte-derived dendritic cells through CXCL10 in cancers. J. Exp. Clin. Cancer Res..

[66] Wu SY (2020). Nicotine promotes brain metastasis by polarizing microglia and suppressing innate immune function. J. Exp. Med..

[67] Yao Z (2018). Imatinib prevents lung cancer metastasis by inhibiting M2-like polarization of macrophages. Pharmacol. Res..

[68] Xiao S (2022). ANGPTL4 regulate glutamine metabolism and fatty acid oxidation in nonsmall cell lung cancer cells. J. Cell Mol. Med..

[69] Zhang K (2021). DNA methylation mediated down-regulation of ANGPTL4 promotes colorectal cancer metastasis by activating the ERK pathway. J. Cancer..

[70] Edlund K (2019). Prognostic Impact of tumor cell programmed death ligand 1 expression and immune cell infiltration in NSCLC. J Thorac Oncol..

[71] Peng DH (2017). ZEB1 induces LOXL2-mediated collagen stabilization and deposition in the extracellular matrix to drive lung cancer invasion and metastasis. Oncogene.

[72] Peng L (2009). Secreted LOXL2 is a novel therapeutic target that promotes gastric cancer metastasis via the Src/FAK pathway. Carcinogenesis.

[73] Tian J (2019). LOXL 2 promotes the epithelial-mesenchymal transition and malignant progression of cervical cancer. Onco Targets Ther..

[74] Salvador F (2017). Lysyl oxidase-like protein LOXL2 promotes lung metastasis of breast cancer. Cancer Res..

[75] Wang C (2019). Lysyl oxidase-like protein 2 promotes tumor lymphangiogenesis and lymph node metastasis in breast cancer. Neoplasia..

[76] Peng DH (2020). Collagen promotes anti-PD-1/PD-L1 resistance in cancer through LAIR1-dependent CD8(+) T cell exhaustion. Nat Commun..

[77] Xie P (2019). Inhibition of LOXL2 enhances the radiosensitivity of castration-resistant prostate cancer cells associated with the reversal of the EMT process. Biomed. Res. Int..

[78] Shen J (2020). PFKP is highly expressed in lung cancer and regulates glucose metabolism. Cell Oncol. (Dordr)..

[79] Zhang L (2021). Hyperbaric oxygen therapy represses the warburg effect and epithelial-mesenchymal transition in hypoxic NSCLC cells via the HIF-1alpha/PFKP axis. Front. Oncol..

[80] Chen J (2022). PFKP alleviates glucose starvation-induced metabolic stress in lung cancer cells via AMPK-ACC2 dependent fatty acid oxidation. Cell Discov..

[81] Lundeen TF (1988). Stress in patients with pain in the muscles of mastication and the temporomandibular joints. J. Oral Rehabil..

[82] Wang Z (2023). PFKP confers chemoresistance by upregulating ABCC2 transporter in non-small cell lung cancer. Transl. Lung Cancer Res..

[83] Mo Z (2020). Identification of a hypoxia-associated signature for lung adenocarcinoma. Front. Genet..

[84] Peng M (2019). Intracellular citrate accumulation by oxidized ATM-mediated metabolism reprogramming via PFKP and CS enhances hypoxic breast cancer cell invasion and metastasis. Cell Death Dis..

[85] Ahluwalia P (2021). Immunogenomic gene signature of cell-death associated genes with prognostic implications in lung cancer. Cancers (Basel).

[86] Chen X (2021). Turning up the heat on non-immunoreactive tumors: Pyroptosis influences the tumor immune microenvironment in bladder cancer. Oncogene..

[87] Zhou N, Bao J (2020). FerrDb: A manually curated resource for regulators and markers of ferroptosis and ferroptosis-disease associations. Database (Oxford)..

