# Supplementary material for: Pan-cell death-related signature reveals tumor immune microenvironment and optimizes personalized therapy alternations in lung adenocarcinoma
Source: Sci Rep. 2024 Jul 8;14:15682. doi: 10.1038/s41598-024-66662-1 (PMC11231366; doi:10.1038/s41598-024-66662-1)

Fig. 9C

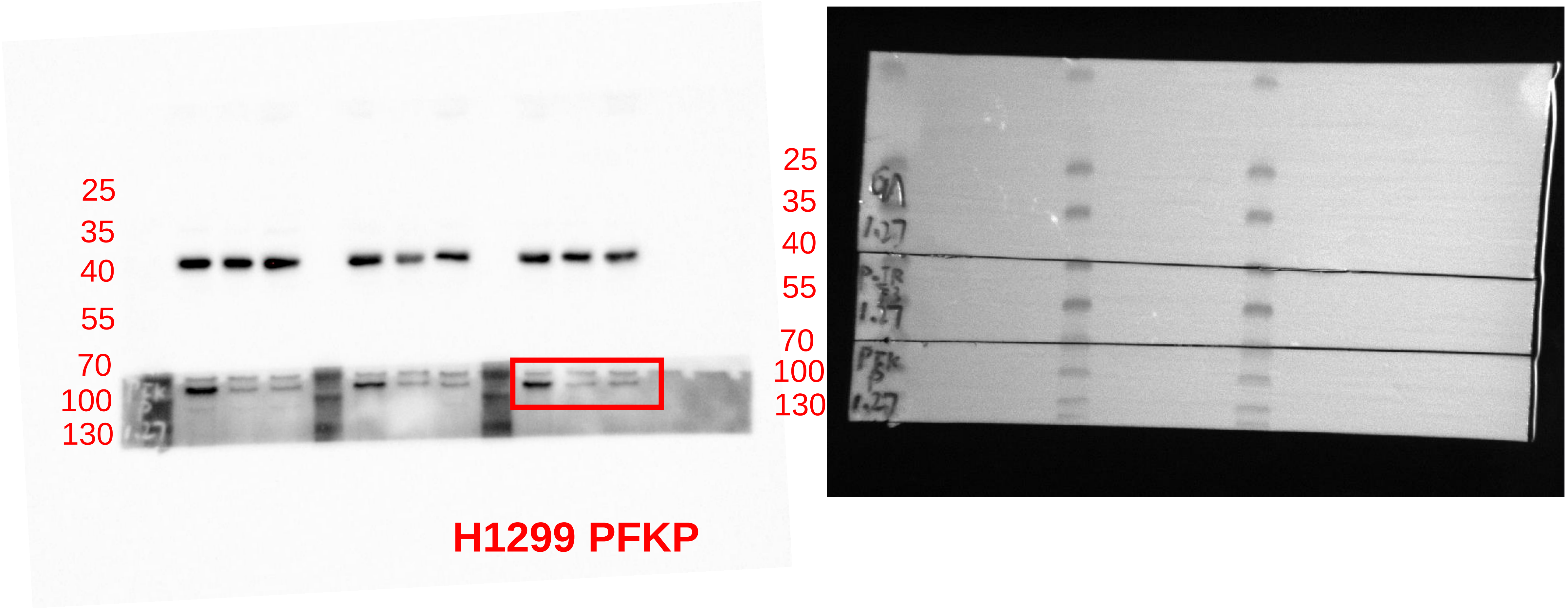

Fig. 9C

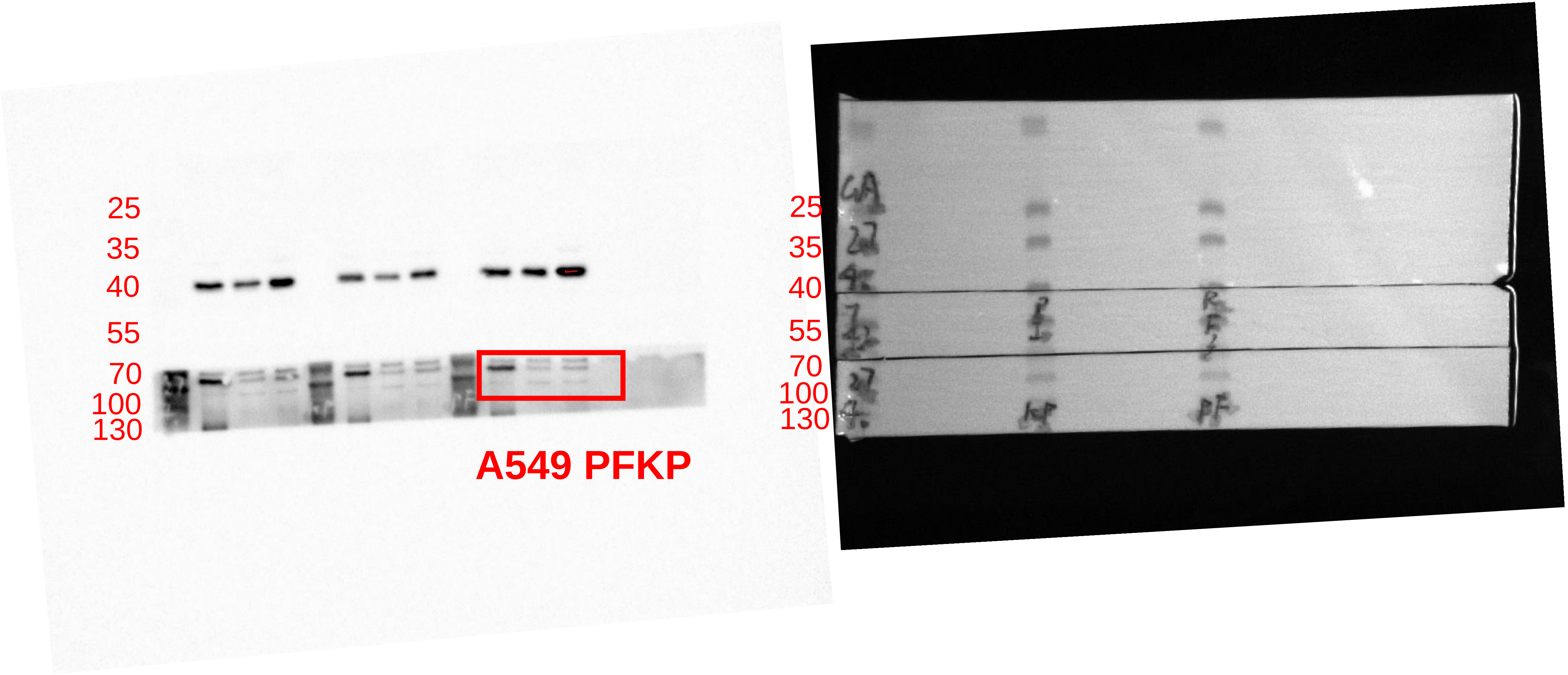

Fig. 9C

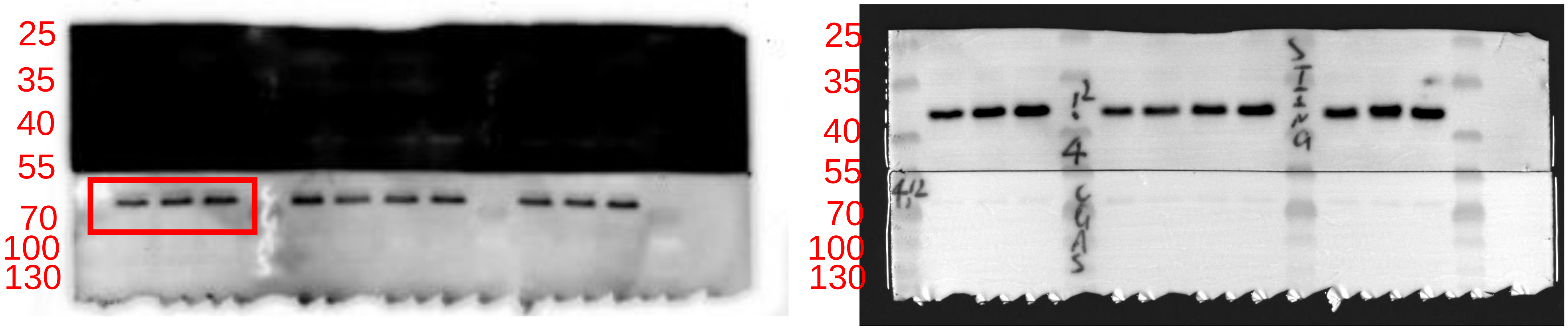

Fig. 9C

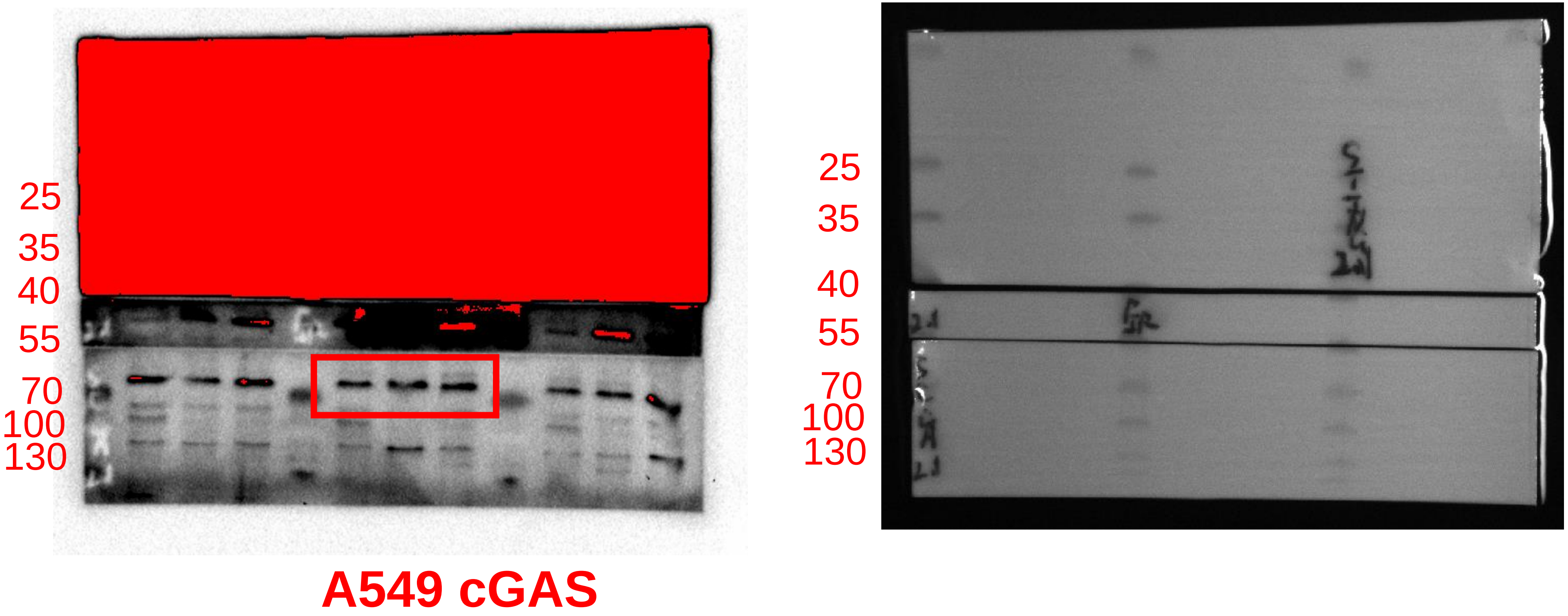

Fig. 9C

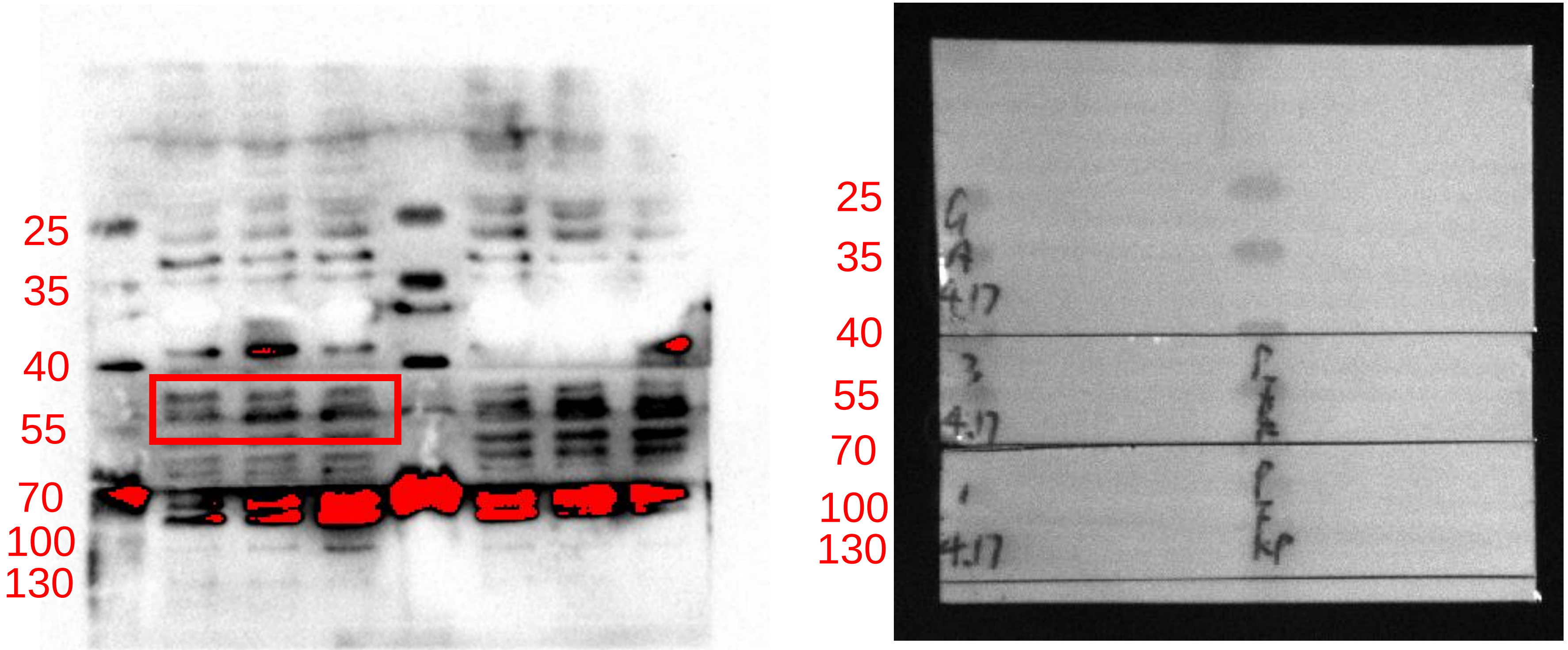

H1299 p-IRF3

Fig. 9C

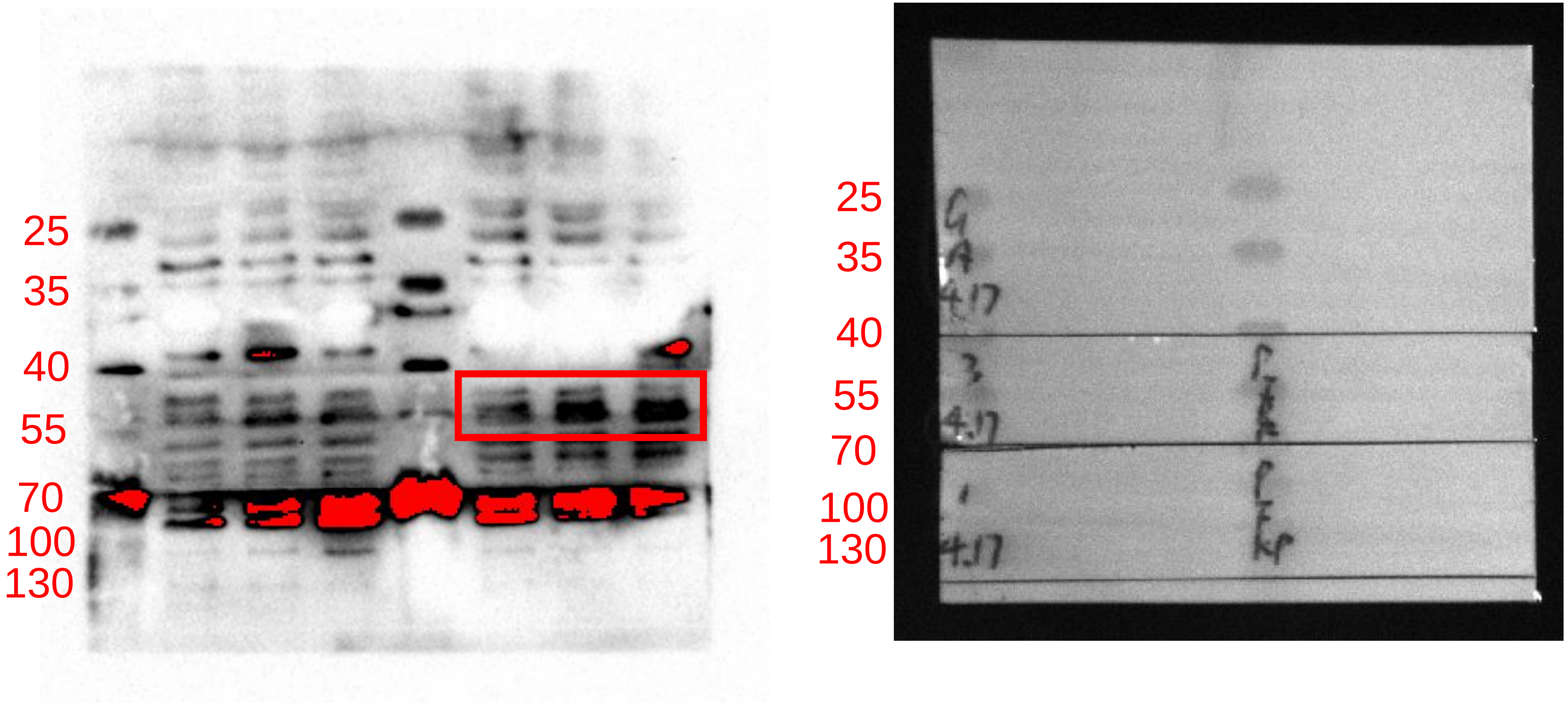

A549 p-IRF3

Fig. 9C

40
55
70

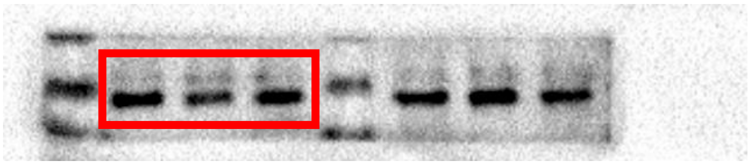

H1299 IRF3

40
55
70

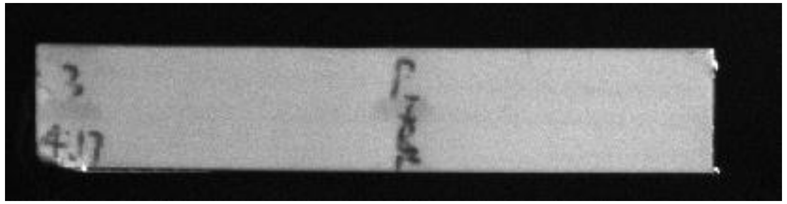

Fig. 9C

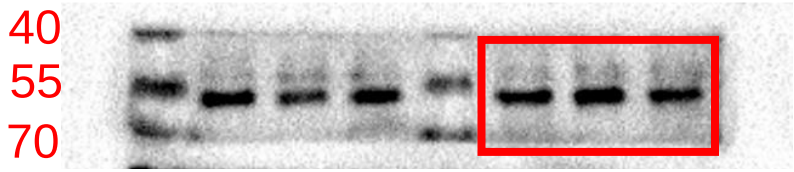

A549 IRF3

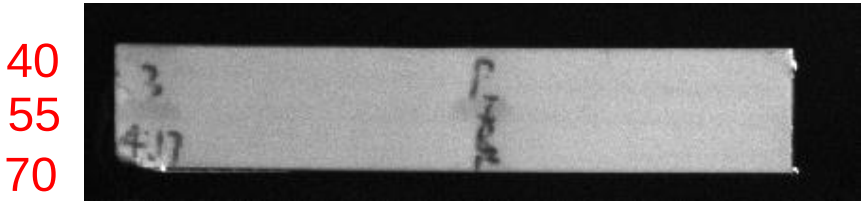

Fig. 9C

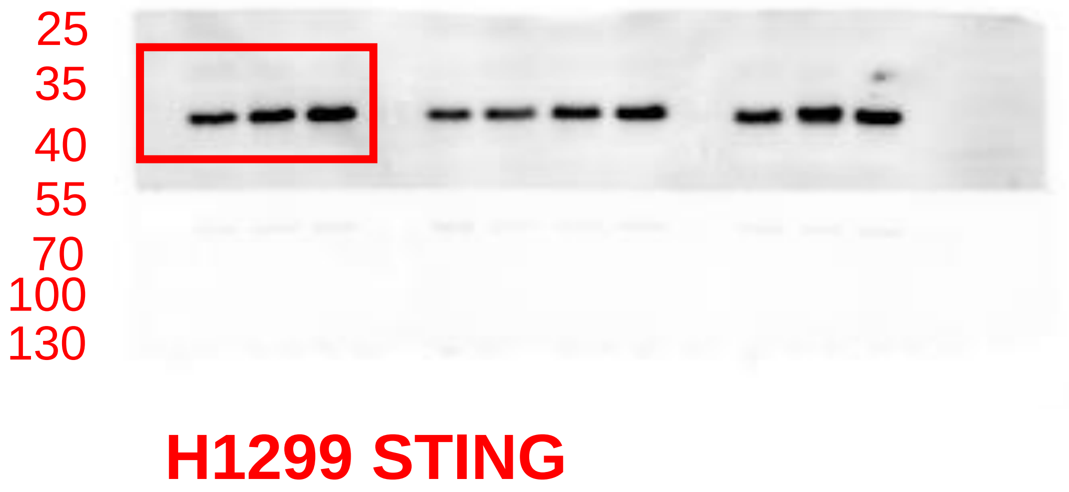

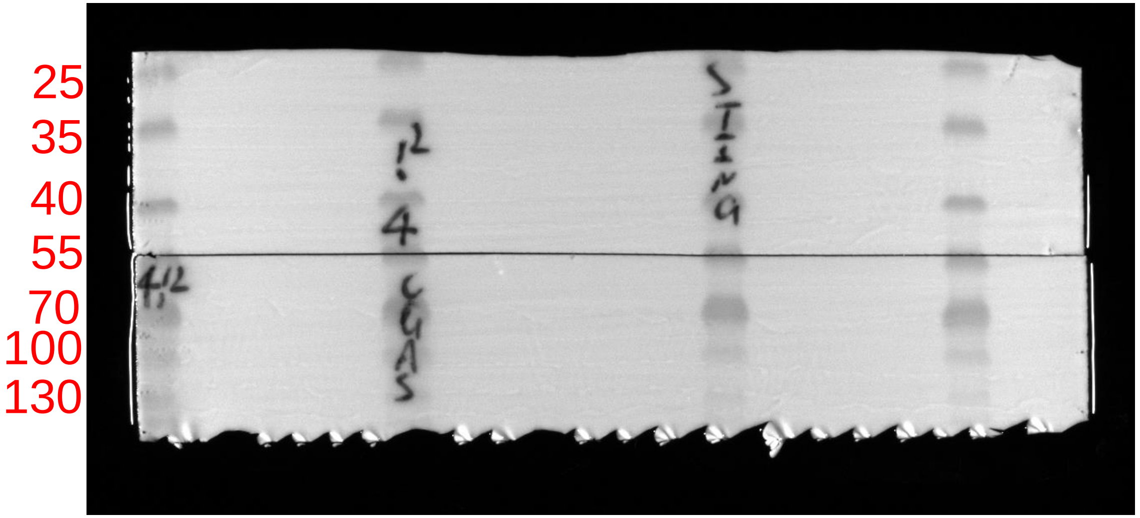

Fig. 9C

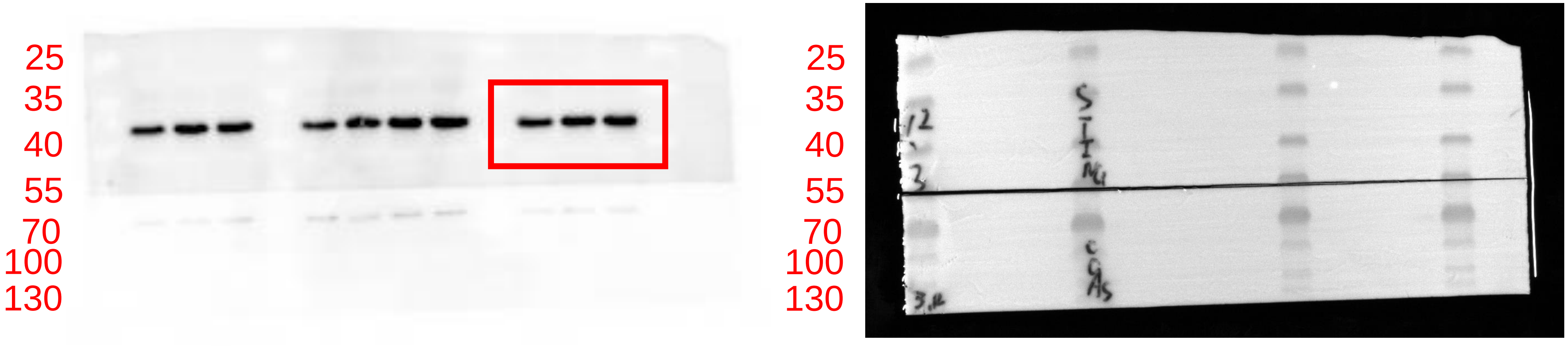

A549 STING

Fig. 9C

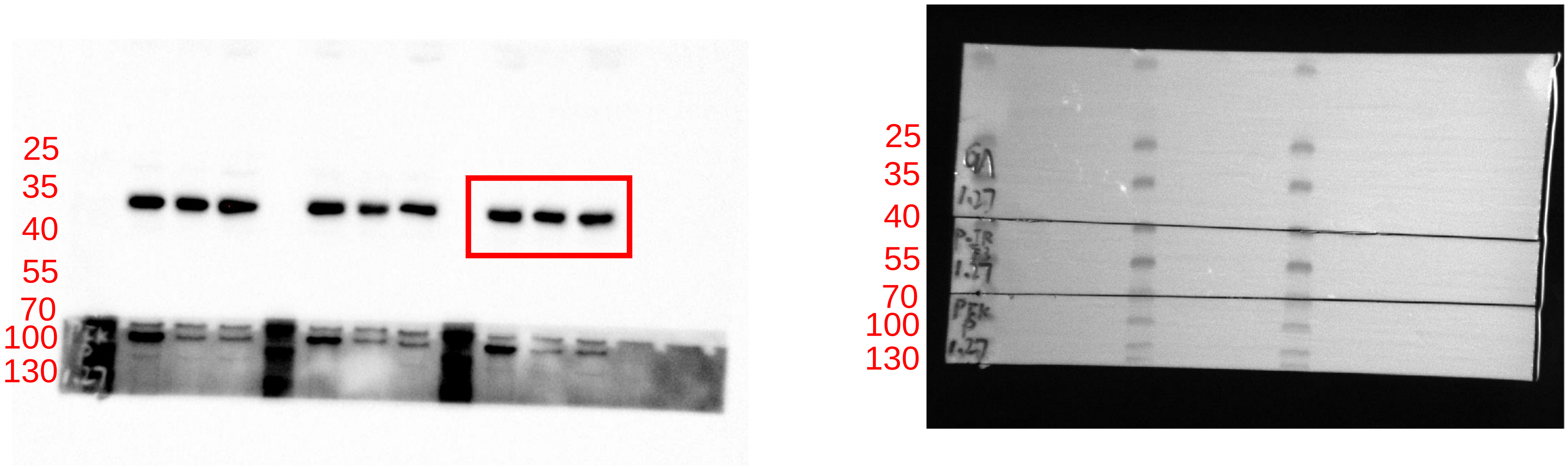

H1299 GAPDH

Fig. 9C

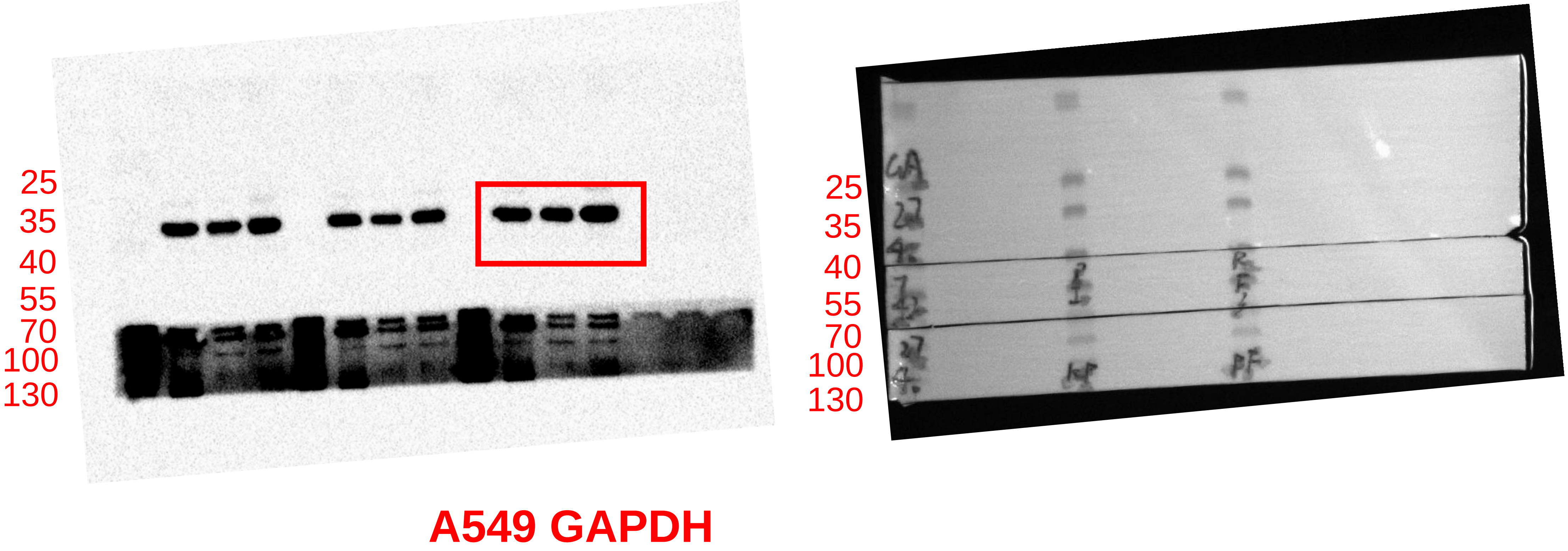

Fig. 10C

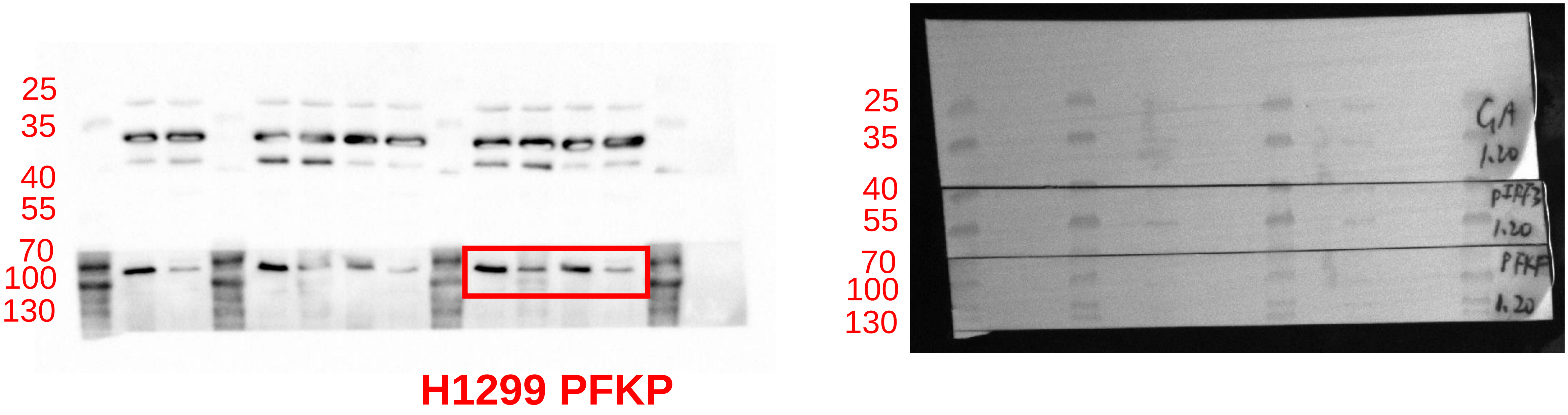

Fig. 10C

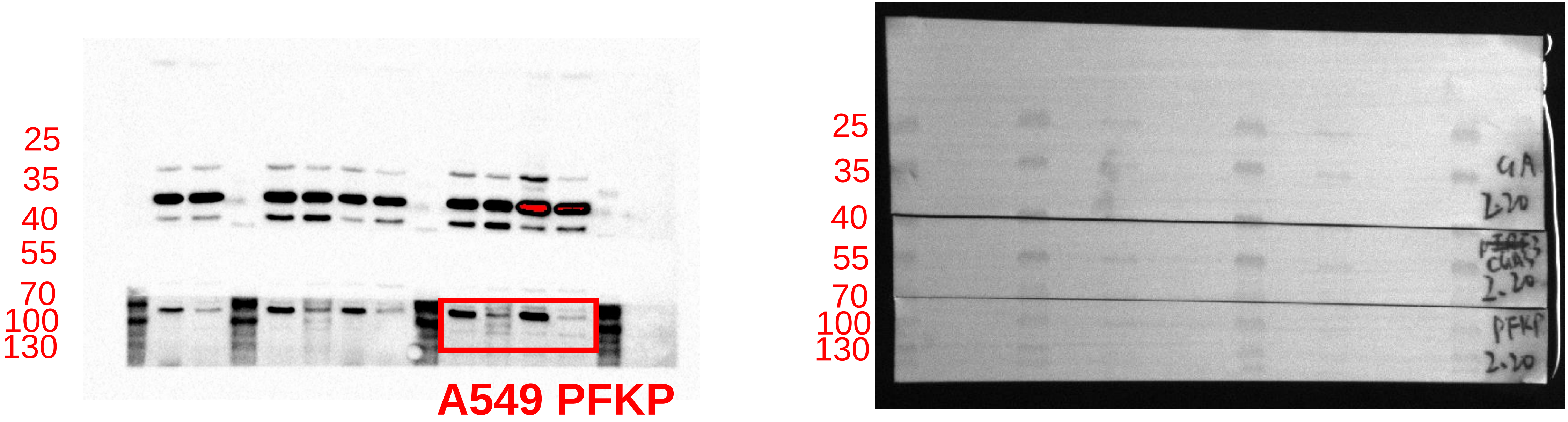

Fig. 10C

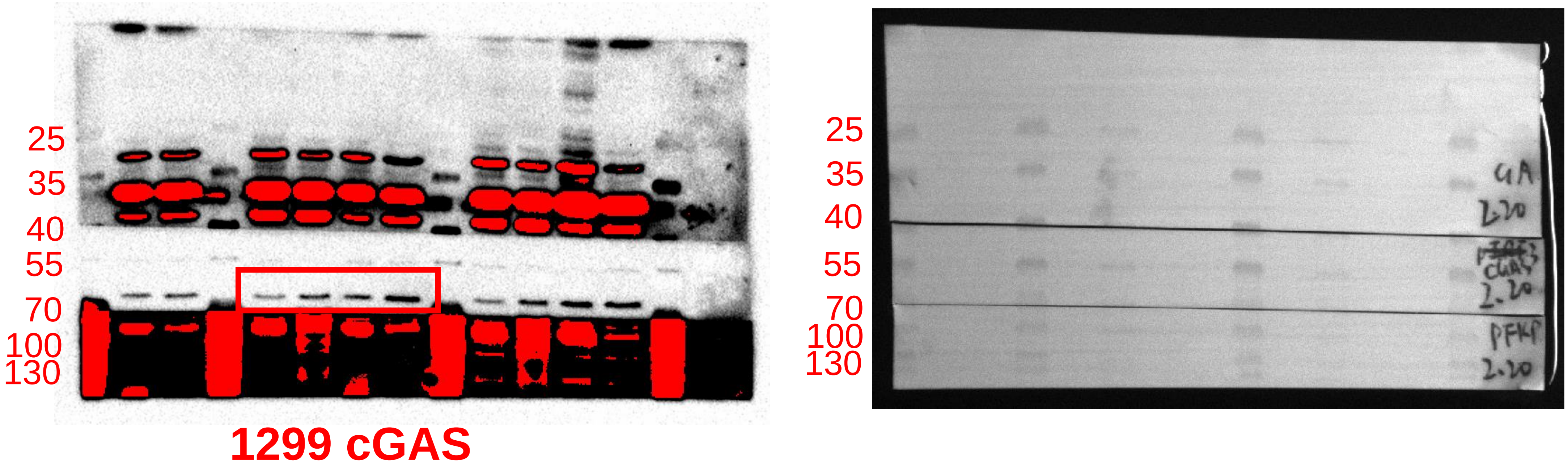

Fig. 10C

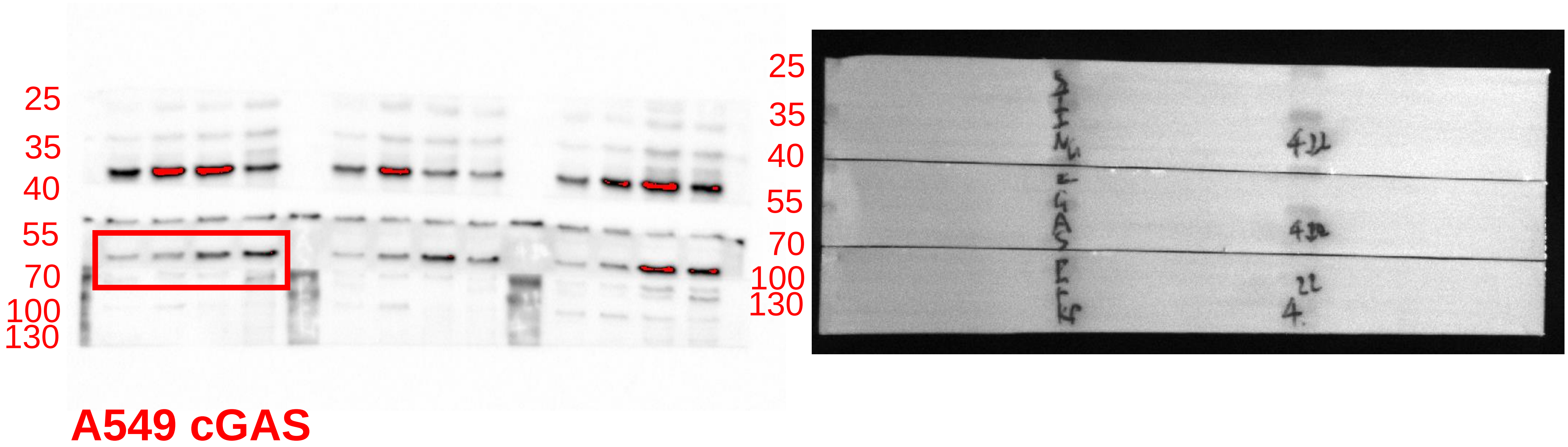

Fig. 10C

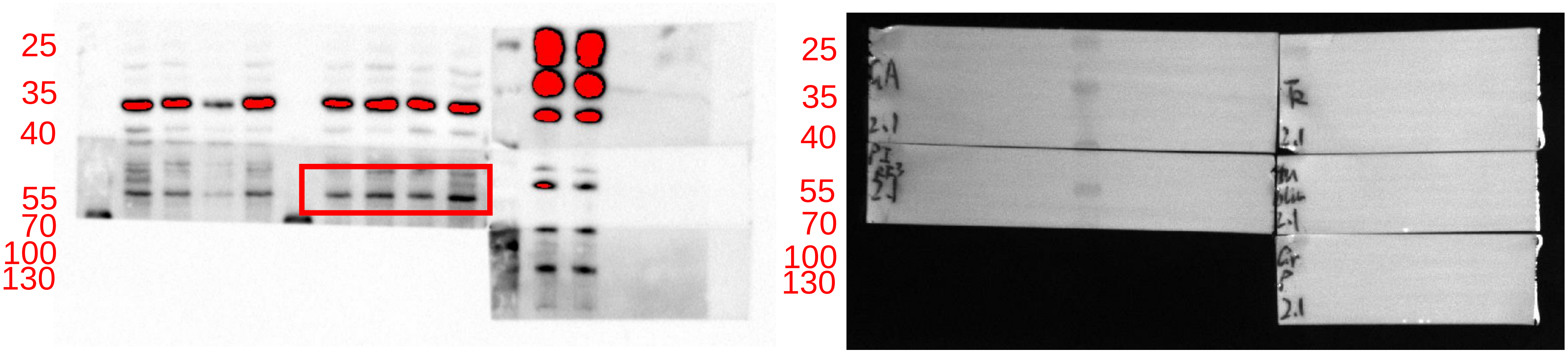

H1299 p-IRF3

Fig. 10C

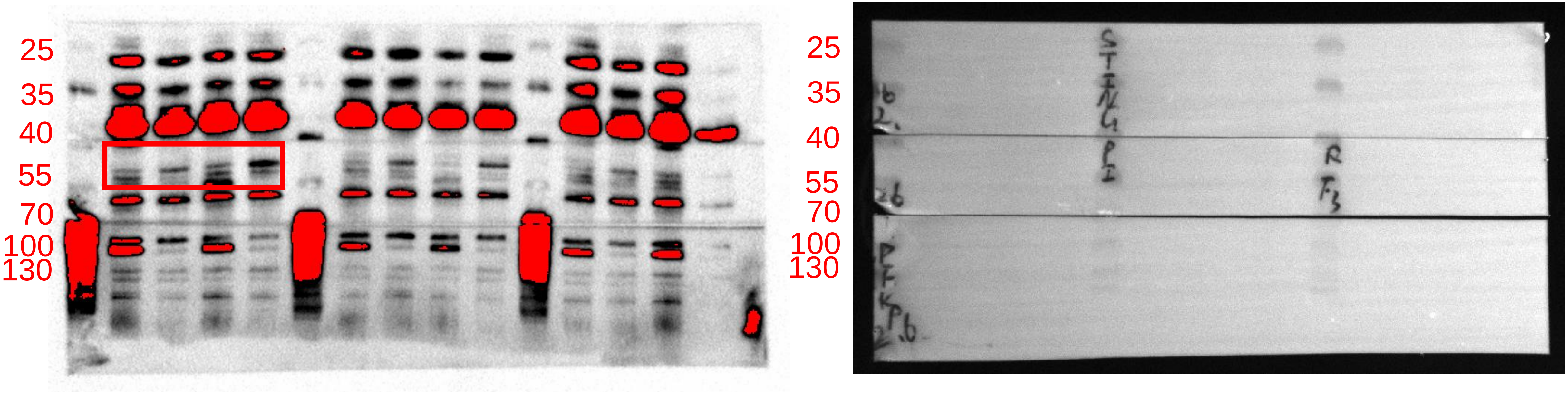

A549 p-IRF3

Fig. 10C

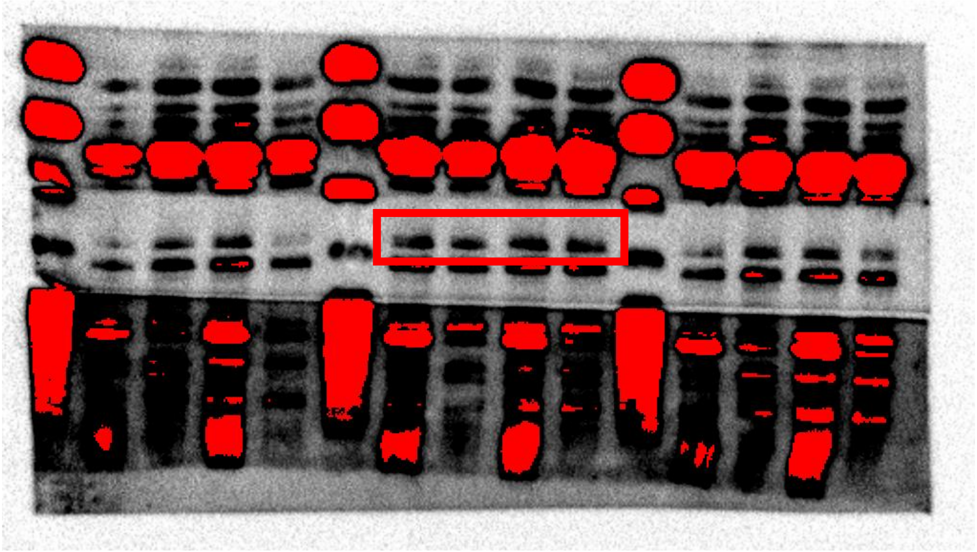

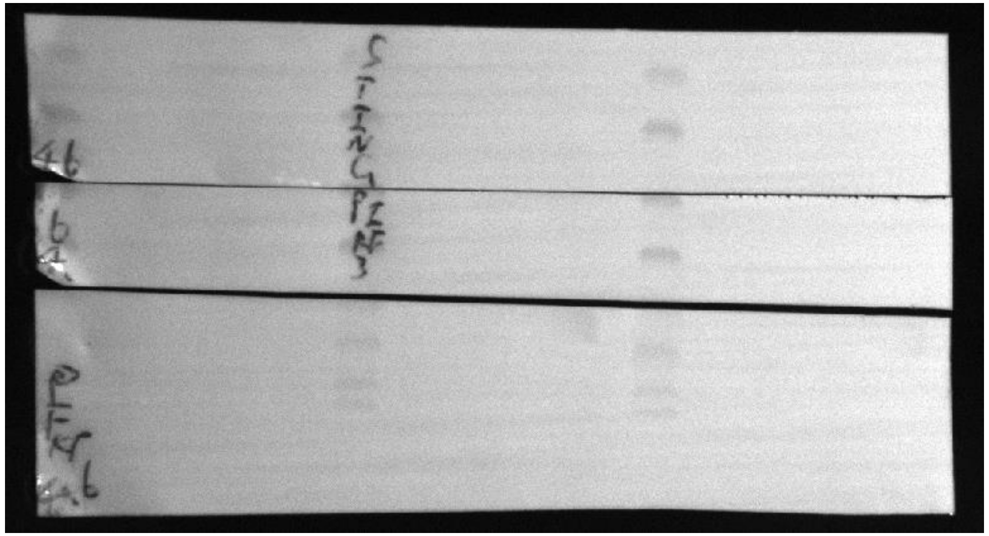

H1299 IRF3

Fig. 10C

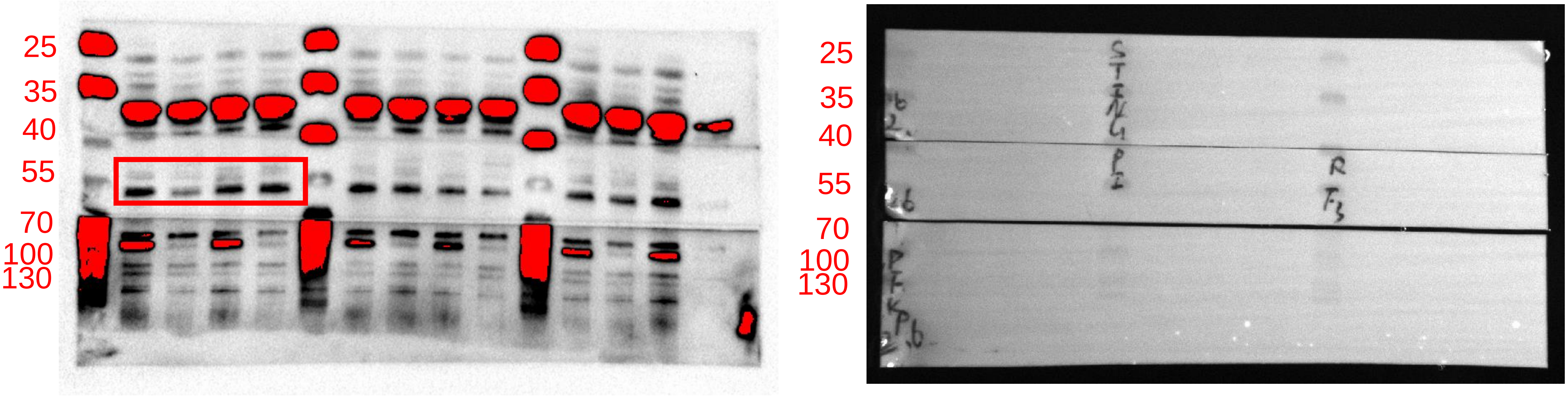

A549 IRF3

Fig. 10C

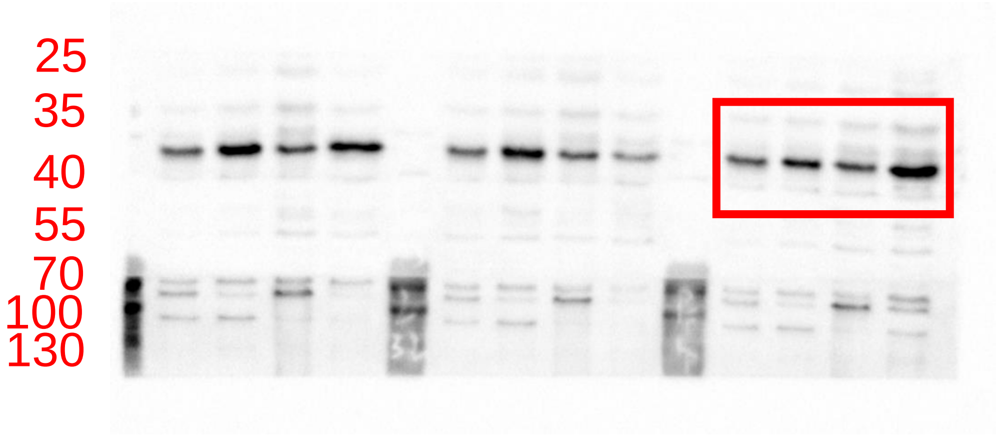

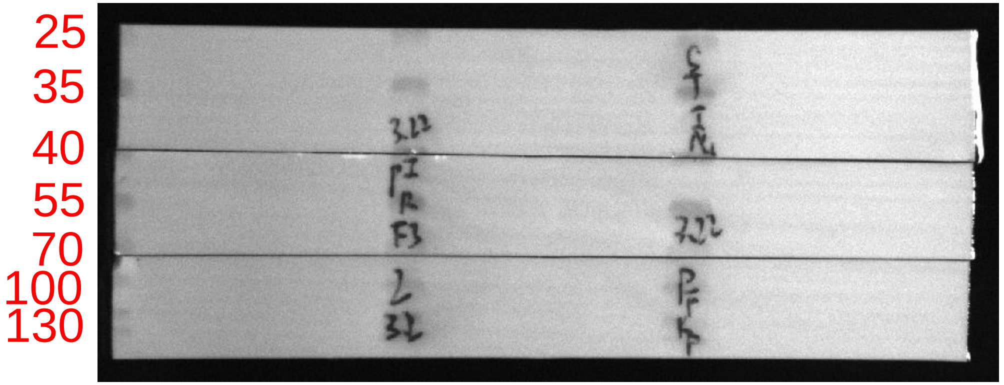

H1299 STING

Fig. 10C

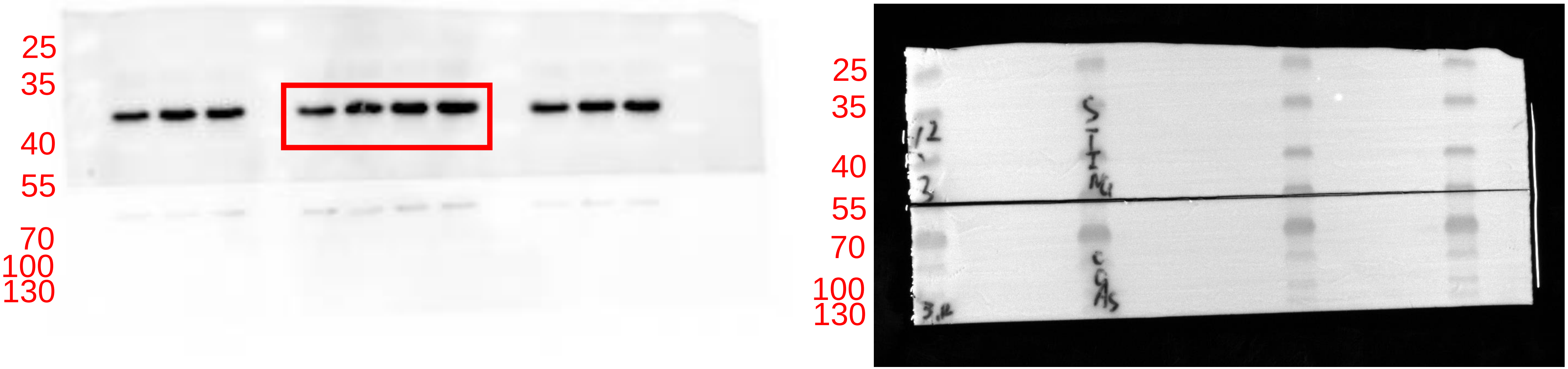

A549 STING

Fig. 10C

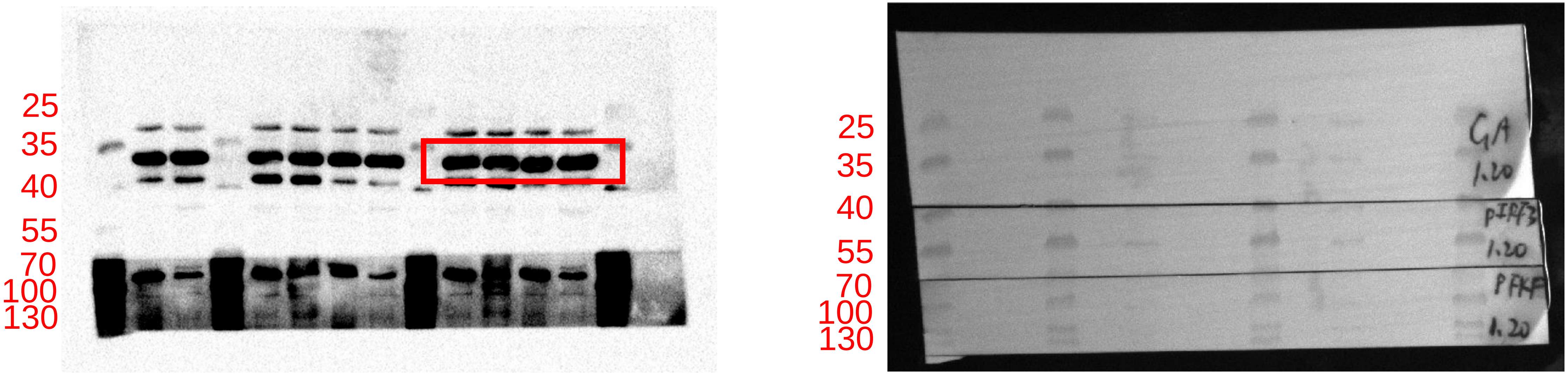

H1299 GAPDH

Fig. 10C

25
35
40
55
70

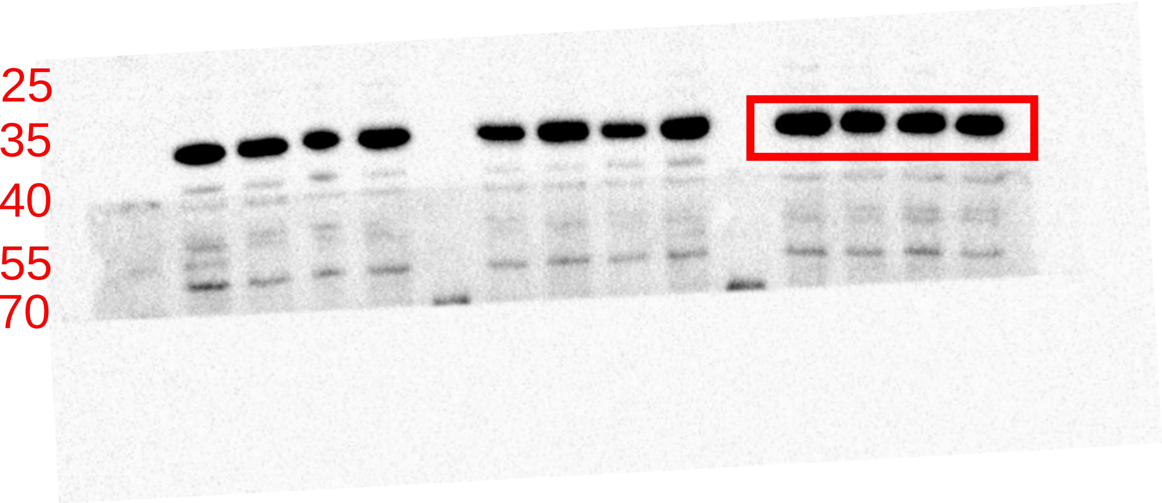

A549 GAPDH

25
35
40
55
70

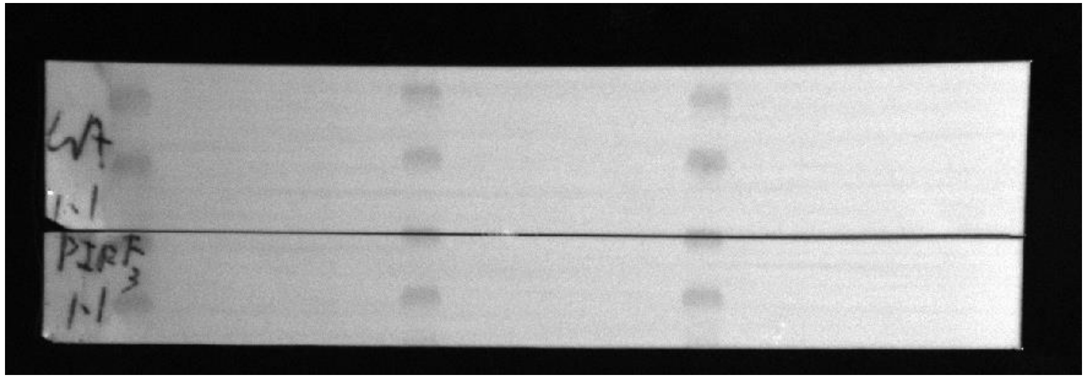

Supplement: Supplementary file 1 — Supplementary Figure 1. [file 41598_2024_66662_MOESM1_ESM.pdf]
