# Supplementary material for: Pan-cell death-related signature reveals tumor immune microenvironment and optimizes personalized therapy alternations in lung adenocarcinoma
Source: Sci Rep. 2024 Jul 8;14:15682. doi: 10.1038/s41598-024-66662-1 (PMC11231366; doi:10.1038/s41598-024-66662-1)
Supplement: Supplementary file 2 — Supplementary Figure 2. [file 41598_2024_66662_MOESM2_ESM.pdf]

Figure S1

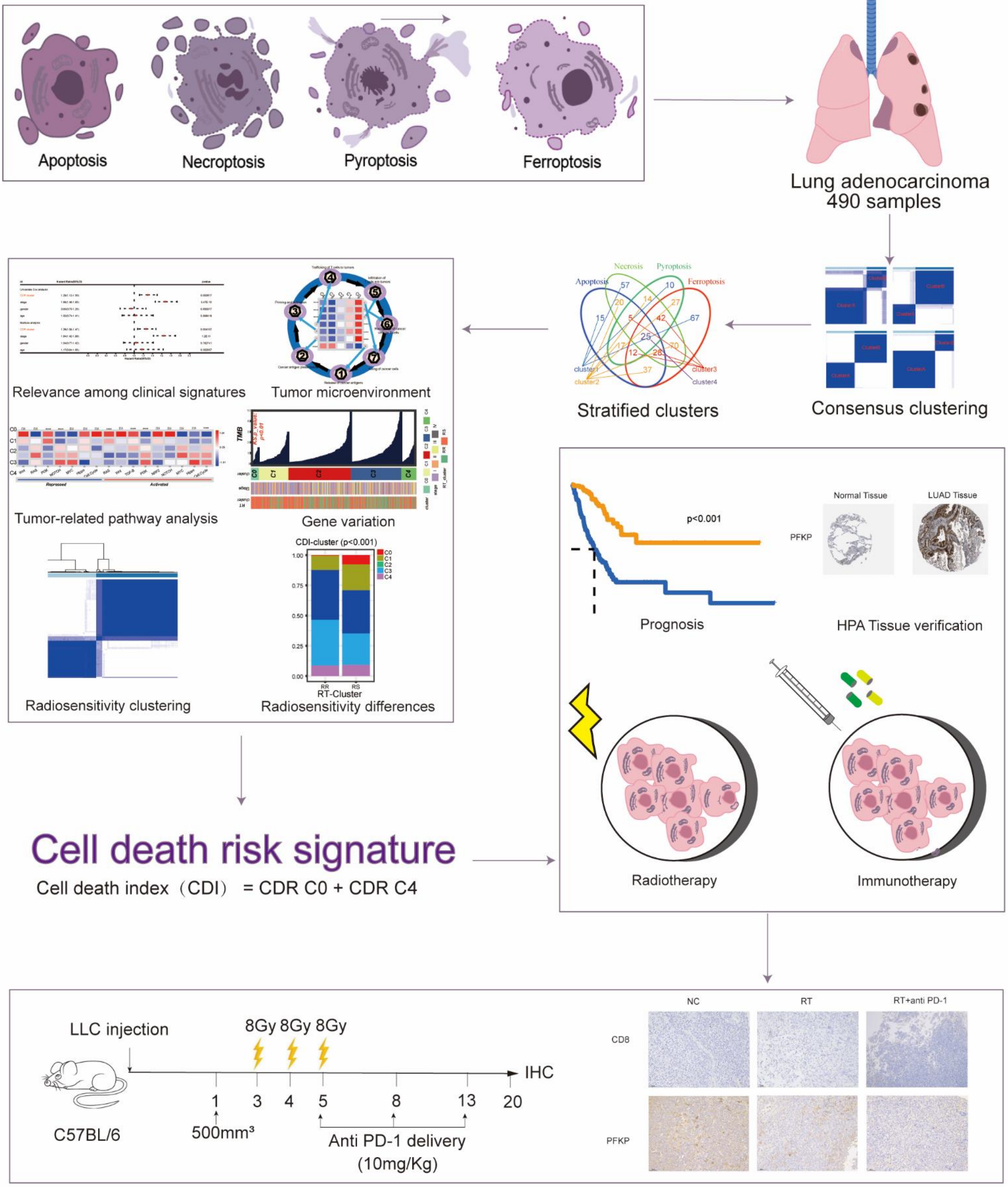

Figure S1. Workflow

Figure S2

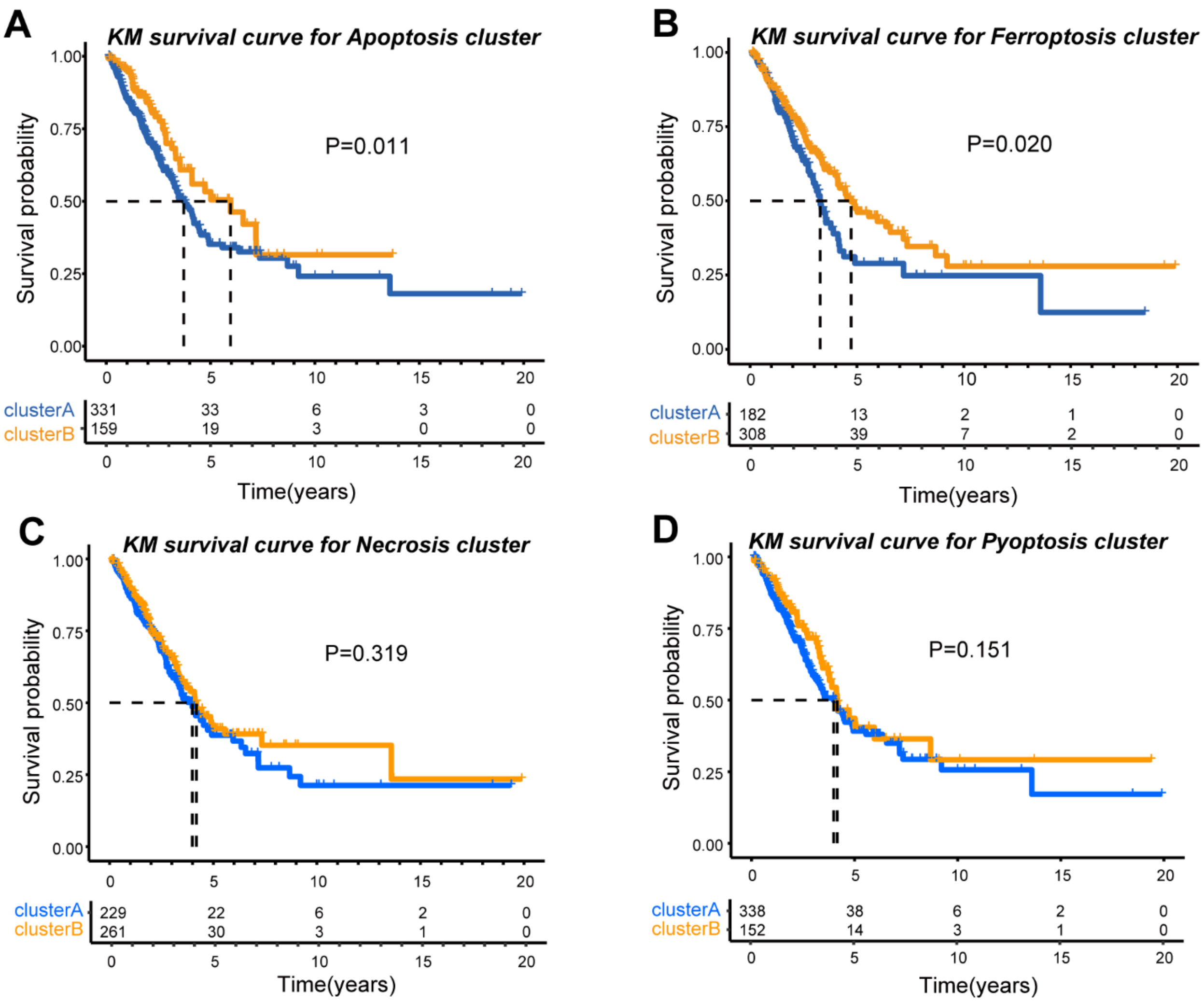

Figure S2. Kaplan–Meier survival curves for four death patterns. A-D Apoptosis (A), Ferroptosis (B), Necrosis (C), Pyroptosis (D).

Figure S3

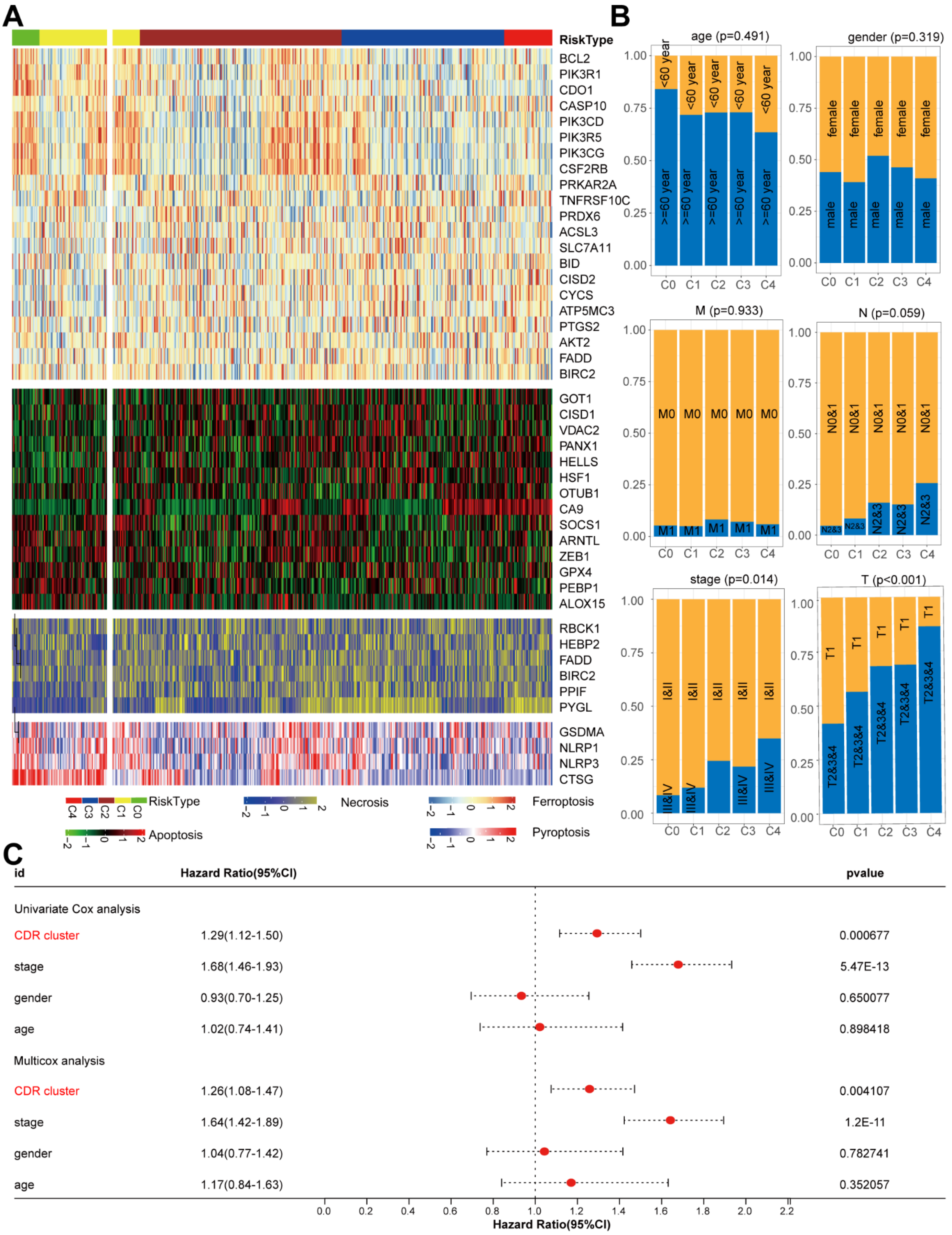

Figure S3. Clinical significance of CDR clusters. A Association between CDR clusters and cell death-related molecules expression variation in composite thermogram (Meta-cohort). **B**

Difference for clinical impactors among five CDR clusters. **C** Univariate and multivariate Cox analysis of the effects of the CDR cluster on clinicopathological factors in the TCGA training set.

Figure S4

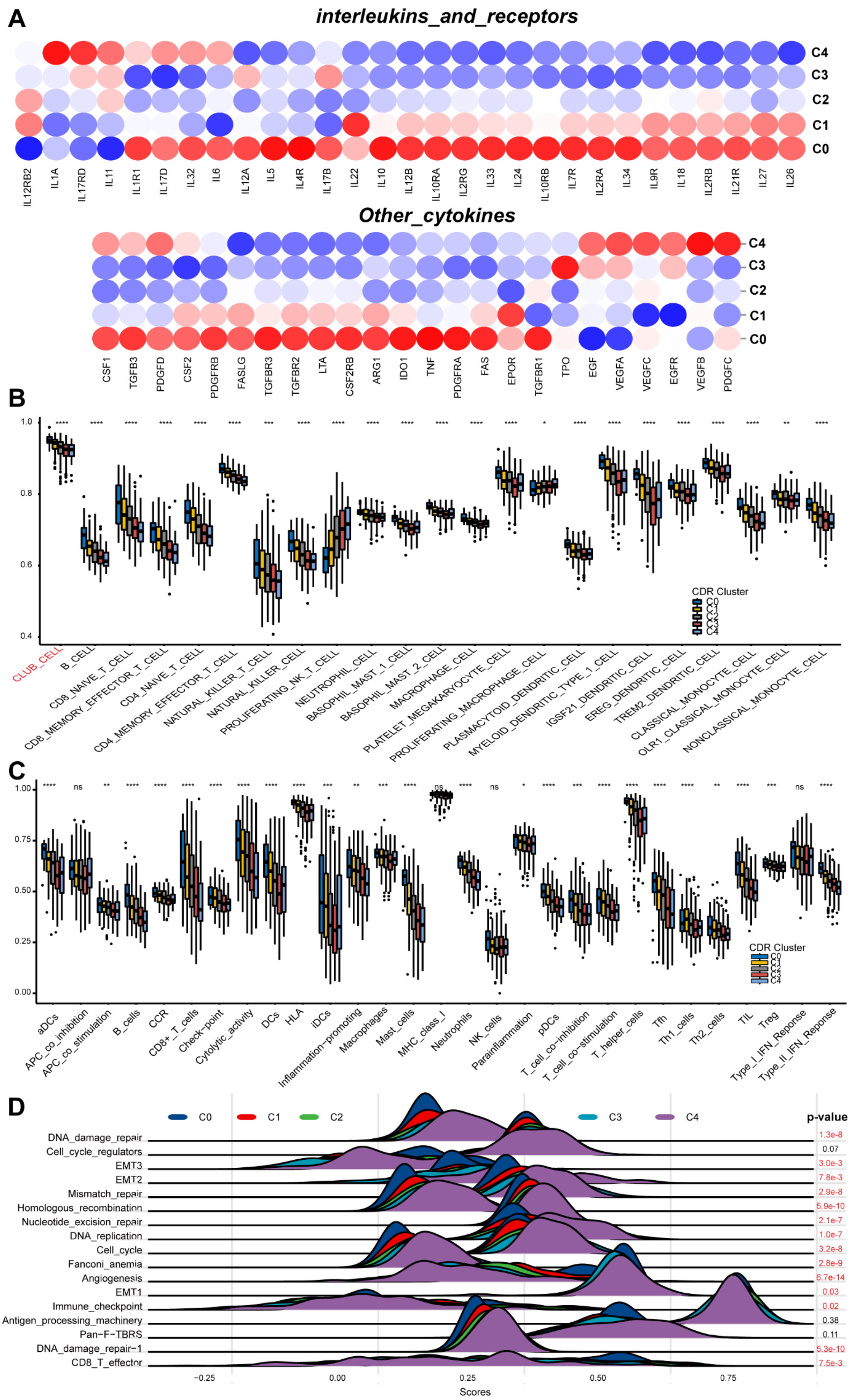

A
interleukins_and_receptors
C4
C3
C2
C1
C0
IL12RB2
IL1A
IL17RD
IL11
IL1R1
IL17D
IL32
IL6
IL12A
IL5
IL4R
IL17B
IL22
IL10
IL12B
IL10RA
IL2RG
IL33
IL24
IL10RB
IL7R
IL2RA
IL34
IL9R
IL18
IL2RB
IL21R
IL27
IL26
Other_cytokines
CSF1
TGFB3
PDGFD
CSF2
PDGFRB
FASLG
TGFBR3
TGFBR2
LTA
CSF2RB
ARG1
IDO1
TNF
PDGFRA
FAS
EPOR
TGFBR1
TPO
EGF
VEGFA
VEGFC
EGFR
VEGFB
PDGFC
B
CDR Cluster
C0
C1
C2
C3
C4
CLUB_CELL
B_CELL
CD8_NAIVE_T_CELL
CD8_MEMORY_EFFECTOR_T_CELL
CD4_NAIVE_T_CELL
CD4_MEMORY_EFFECTOR_T_CELL
NATURAL_KILLER_T_CELL
NATURAL_KILLER_CELL
PROLIFERATING_NK_T_CELL
NEUTROPHIL_CELL
BASOPHIL_MAST_1_CELL
BASOPHIL_MAST_2_CELL
MACROPHAGE_CELL
PLATELET_MEGAKARYOCYTE_CELL
PROLIFERATING_MACROPHAGE_CELL
PLASMACYTOID_DENDRITIC_CELL
MYELOID_DENDRITIC_TYPE_1_CELL
IGSF21_DENDRITIC_CELL
EREG_DENDRITIC_CELL
TREM2_DENDRITIC_CELL
CLASSICAL_MONOCYTE_CELL
OLR1_CLASSICAL_MONOCYTE_CELL
NONCLASSICAL_MONOCYTE_CELL
C
CDR Cluster
aDCs
APC_co_inhibition
APC_co_stimulation
B_cells
CCR
CD8+_T_cells
Check-point
Cytolytic_activity
DCs
HLA
iDCs
Inflammation-promoting
Macrophages
Mast_cells
MHC_class_I
Neutrophils
NK_cells
Parainflammation
pDCs
T_cell_co-inhibition
T_cell_co-stimulation
T_helper_cells
Tfh
Th1_cells
Th2_cells
TIL
Treg
Type_I_IFN_Reponse
Type_II_IFN_Reponse
D
C0
C1
C2
C3
C4
p-value
DNA_damage_repair 1.3e-8
Cell_cycle_regulators 0.07
EMT3 3.0e-3
EMT2 7.8e-3
Mismatch_repair 2.9e-8
Homologous_recombination 5.9e-10
Nucleotide_excision_repair 2.1e-7
DNA_replication 1.0e-7
Cell_cycle 3.2e-8
Fanconi_anemia 2.8e-9
Angiogenesis 6.7e-14
EMT1 0.03
Immune_checkpoint 0.02
Antigen_processing_machinery 0.38
Pan-F-TBRS 0.11
DNA_damage_repair-1 5.3e-10
CD8_T_effector 7.5e-3
Scores

Figure S4. The level of interleukins, receptors, cytokines and immune cells infiltration among CDR clusters. A Expression of interleukins, receptors and cytokines in CDR patients. **B-C** Comparison of immune cells infiltration among CDR clusters. **D** Difference of immune-related pathways among CDR clusters.

Figure S5

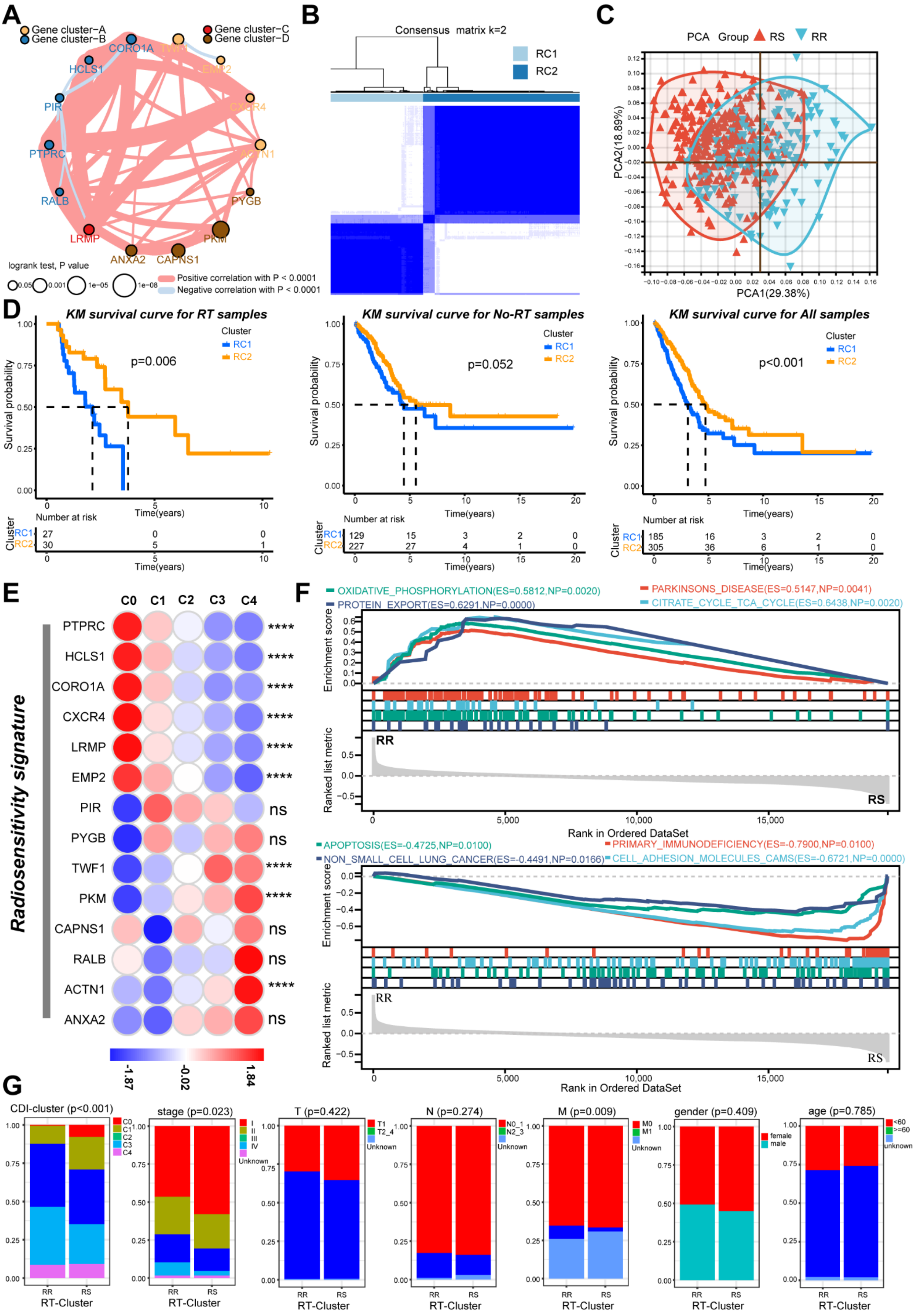
A
Gene cluster−A
Gene cluster−B
Gene cluster−C
Gene cluster−D
CORO1A
TWF1
HCLS1
EMP2
PIR
CXCR4
PTPRC
ACTN1
RALB
PYGB
LRMP
PKM
ANXA2
CAPNS1
logrank test, P value
0.05
0.001
1e−05
1e−08
Positive correlation with P < 0.0001
Negative correlation with P < 0.0001
B
Consensus matrix k=2
RC1
RC2
C
PCA Group RS RR
PCA2(18.89%)
PCA1(29.38%)
D
KM survival curve for RT samples
Cluster
RC1
RC2
p=0.006
Survival probability
Time(years)
Number at risk
RC1 27 0 0
RC2 30 5 1
KM survival curve for No-RT samples
p=0.052
RC1 129 15 3 2 0
RC2 227 27 4 1 0
KM survival curve for All samples
p<0.001
RC1 185 16 3 2 0
RC2 305 36 6 1 0
E
C0 C1 C2 C3 C4
Radiosensitivity signature
PTPRC ****
HCLS1 ****
CORO1A ****
CXCR4 ****
LRMP ****
EMP2 ****
PIR ns
PYGB ns
TWF1 ****
PKM ****
CAPNS1 ns
RALB ns
ACTN1 ****
ANXA2 ns
-1.87
-0.02
1.84
F
OXIDATIVE_PHOSPHORYLATION(ES=0.5812,NP=0.0020)
PROTEIN_EXPORT(ES=0.6291,NP=0.0000)
PARKINSONS_DISEASE(ES=0.5147,NP=0.0041)
CITRATE_CYCLE_TCA_CYCLE(ES=0.6438,NP=0.0020)
Enrichment score
Ranked list metric
RR
RS
Rank in Ordered DataSet
APOPTOSIS(ES=-0.4725,NP=0.0100)
NON_SMALL_CELL_LUNG_CANCER(ES=-0.4491,NP=0.0166)
PRIMARY_IMMUNODEFICIENCY(ES=-0.7900,NP=0.0100)
CELL_ADHESION_MOLECULES_CAMS(ES=-0.6721,NP=0.0000)
G
CDI-cluster (p<0.001)
C0 C1 C2 C3 C4
stage (p=0.023)
I II III IV Unknown
T (p=0.422)
T1 T2_4 Unknown
N (p=0.274)
N0_1 N2_3 Unknown
M (p=0.009)
M0 M1 Unknown
gender (p=0.409)
female male
age (p=0.785)
<60 >=60 unknown
RR RS
RT-Cluster

Figure S5. Correlation between RT sensitivity and CDR clusters. A Radiosensitive (RS)-related genes in LUAD patients. **B** Consensus clustering for LUAD patients via RS-related genes. **C** PCA analysis for RR and RS samples. **D** Kaplan–Meier survival curves for samples with RT (left), No-RT (middle) and all samples (right). **E** Expression level of radiosensitive genes amone CDR clusters. **F** Enriched pathways in RR and RS group. **G** Rate of CDR cluster, stage, T stage, N stage, M stage, gender and age in the RR and RS group. (*P < 0.05, ** P < 0.01, *** P < 0.001, ****P < 0.0001, ns P > 0.05).

Figure S6

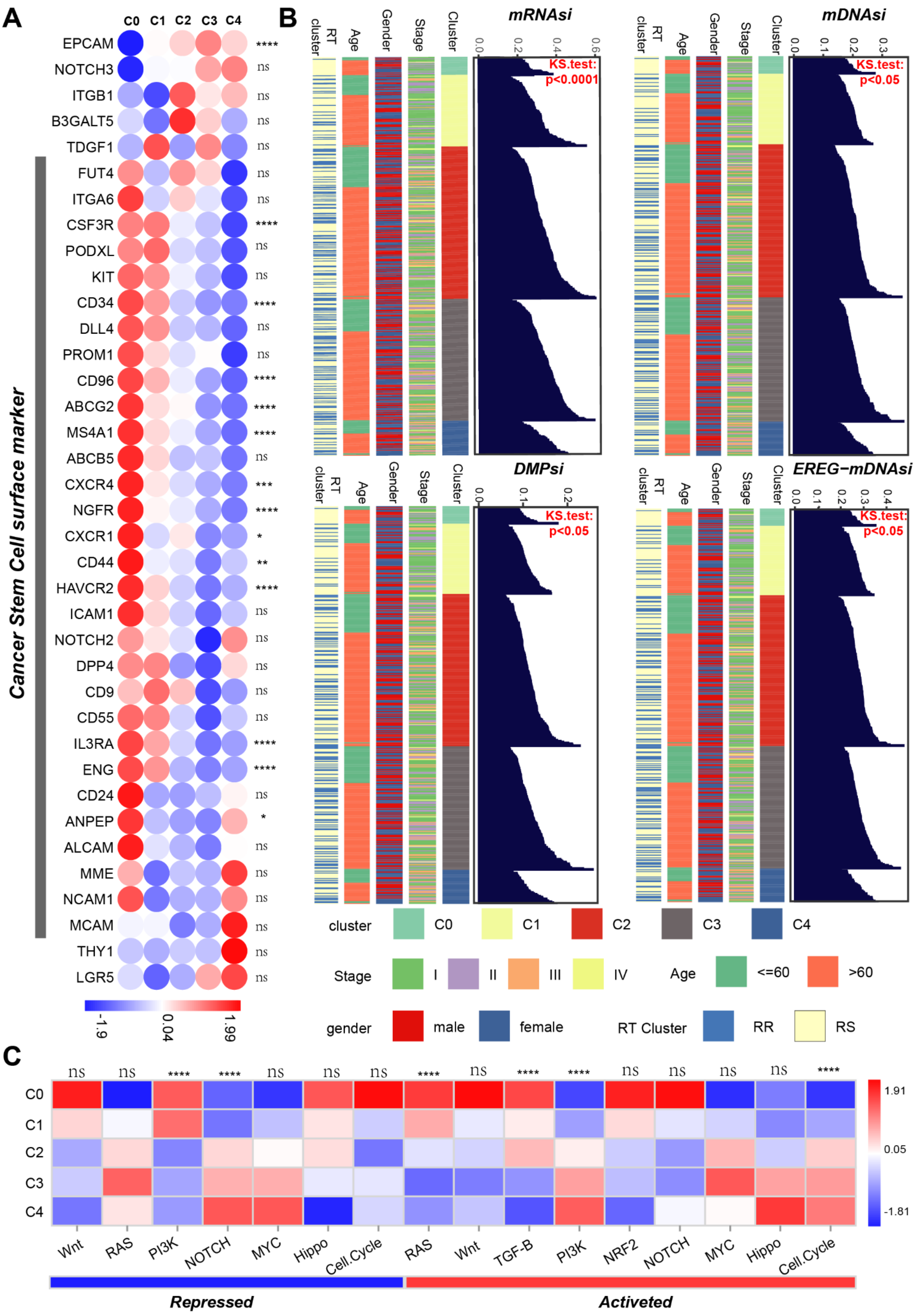

A
C0 C1 C2 C3 C4
EPCAM ****
NOTCH3 ns
ITGB1 ns
B3GALT5 ns
TDGF1 ns
FUT4 ns
ITGA6 ns
CSF3R ****
PODXL ns
KIT ns
CD34 ****
DLL4 ns
PROM1 ns
CD96 ****
ABCG2 ****
MS4A1 ****
ABCB5 ns
CXCR4 ***
NGFR ****
CXCR1 *
CD44 **
HAVCR2 ****
ICAM1 ns
NOTCH2 ns
DPP4 ns
CD9 ns
CD55 ns
IL3RA ****
ENG ****
CD24 ns
ANPEP *
ALCAM ns
MME ns
NCAM1 ns
MCAM ns
THY1 ns
LGR5 ns
Cancer Stem Cell surface marker
-1.9
0.04
1.99
B
RT cluster
Age
Gender
Stage
Cluster
mRNAsi
KS.test: p<0.0001
mDNAsi
KS.test: p<0.05
DMPsi
KS.test: p<0.05
EREG−mDNAsi
KS.test: p<0.05
cluster C0 C1 C2 C3 C4
Stage I II III IV
Age <=60 >60
gender male female
RT Cluster RR RS
C
ns ns **** **** ns ns ns **** ns **** **** ns ns ns ns ****
C0
C1
C2
C3
C4
1.91
0.05
-1.81
Wnt RAS PI3K NOTCH MYC Hippo Cell.Cycle RAS Wnt TGF-B PI3K NRF2 NOTCH MYC Hippo Cell.Cycle
Repressed
Activeted

D

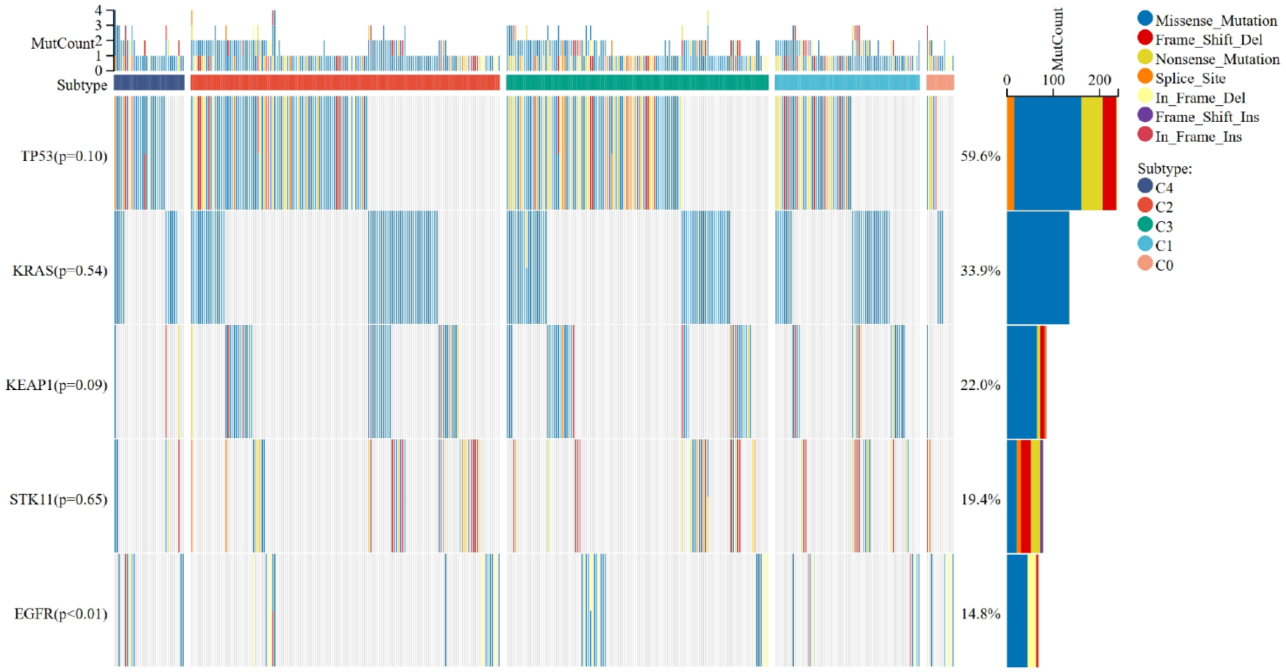

Figure S6. Cancer stemness characteristic and driver mutation alternations of CDR clusters. A Cancer stem cell surface marker expression among CDR clusters. **B** Correlation of CDR clusters and clinical features in mRNAsi/mDNAsi/DMPsi/EREG-mDNAsi. **C** Known tumorigenic pathways variation in CDR clusters. **D** Driver mutation alternations among CDR clusters. (complete response [CR], stable disease [SD], progressive disease [PD]; Chi-square test, p = 0.046). (****P < 0.0001, ns P > 0.05).

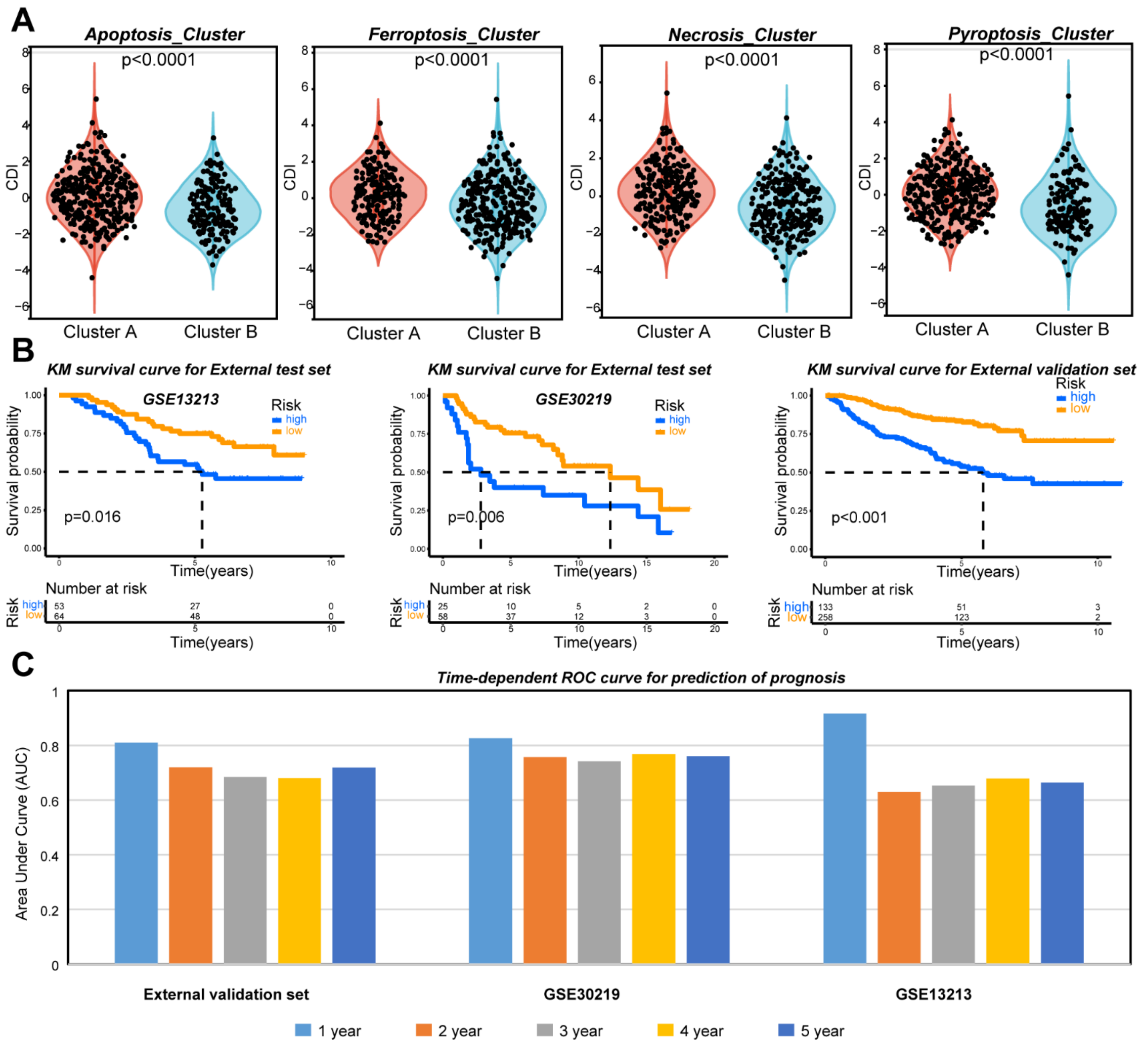

Figure S7. Validation of correlation between CDI and Cluster A&B in death patterns. A The Kruskal–Wallis test evaluates the CDIScore of different patterns. **B** Kaplan–Meier survival curves for test and validation sets stratified by high and low-risk CDRSig. **C** ROC curve for test and validation sets.

Figure S8

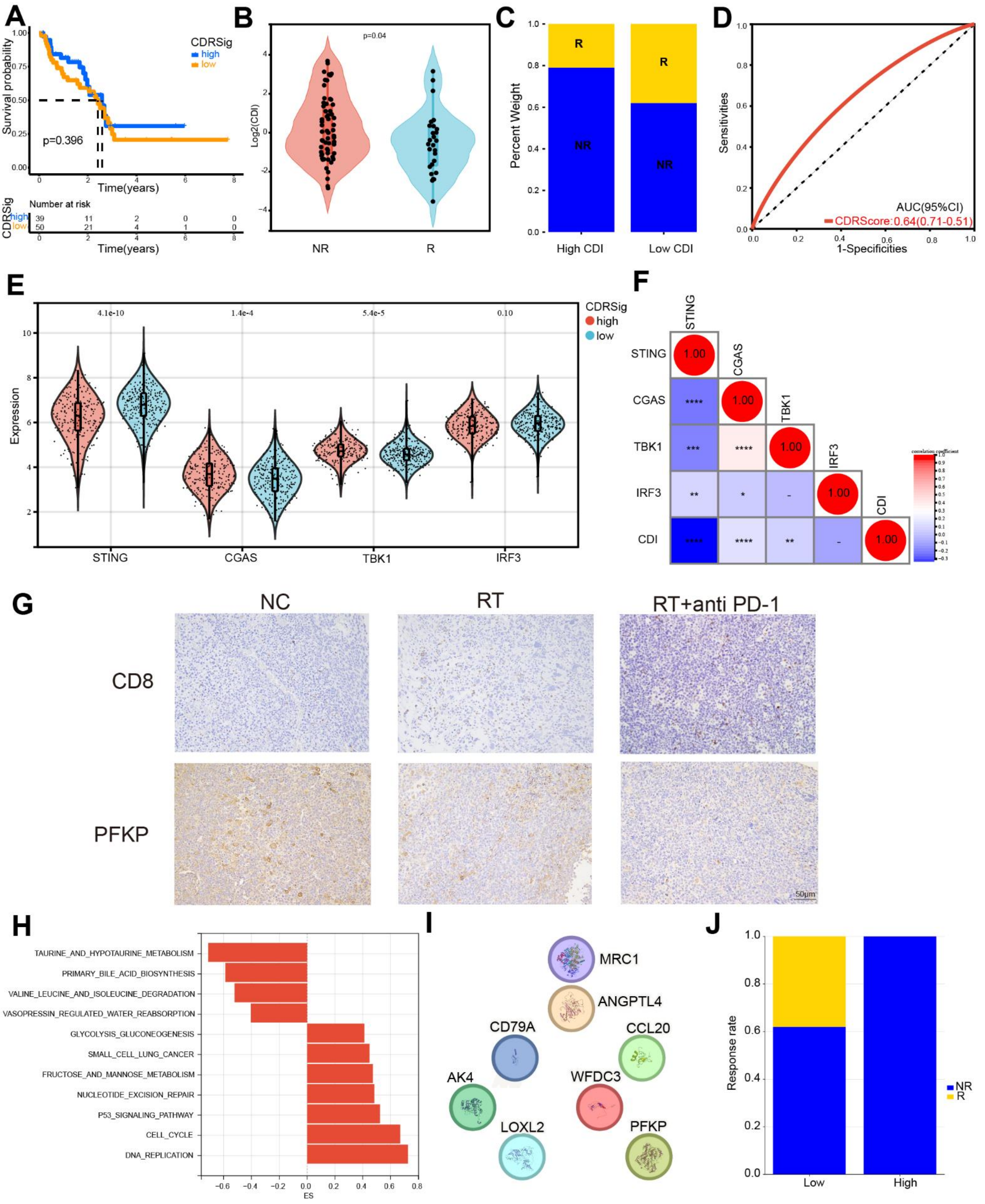

Figure S8. Prediction of prognosis and cGAS/STING signaling pathway activation. A Survival curve of CDRSig in GSE 30219. **B** Correlation of CDI between tumor relapse and no-relapse. **C** Rate of relapse in high CDI and low CDI. **D** ROC curve of CDIScore in radiosensitivity prediction. **E, F** Expression of cGAS/STING-related genes. **G** Representative IHC of CD8, PFKP

staining in three xenograft model groups. **H** GSEA for PFKP in LUAD. **I** Protein-protein interaction networks (STRING) for genes in CDRSig. **J** Response to anti-PD1 immunotherapy in high and low CDI group.
